# Electrospraying as a Technique for the Controlled Synthesis of Biocompatible PLGA@Ag_2_S and PLGA@Ag_2_S@SPION Nanocarriers with Drug Release Capability

**DOI:** 10.3390/pharmaceutics14010214

**Published:** 2022-01-17

**Authors:** Alexis Alvear-Jiménez, Irene Zabala Gutierrez, Yingli Shen, Gonzalo Villaverde, Laura Lozano-Chamizo, Pablo Guardia, Miguel Tinoco, Beatriz Garcia-Pinel, José Prados, Consolación Melguizo, Manuel López-Romero, Daniel Jaque, Marco Filice, Rafael Contreras-Cáceres

**Affiliations:** 1 Departamento de Química en Ciencias Farmacéuticas, Universidad Complutense de Madrid, 28040 Madrid, Spain; alexial@ucm.es (A.A.-J.); irenezab@ucm.es (I.Z.G.); gonvilla@ucm.es (G.V.); 2 Fluorescence Imaging Group, Facultad de Ciencias, Universidad Autónoma de Madrid, 28049 Madrid, Spain; yingli.shen@estudiante.uam.es (Y.S.); daniel.jaque@uam.es (D.J.); 3 Nanobiotechnology for Life Sciences Group, Department of Chemistry in Pharmaceutical Sciences, Faculty of Pharmacy, Universidad Complutense de Madrid (UCM), Plaza Ramón y Cajal, 28040 Madrid, Spain; laurloza@ucm.es (L.L.-C.); mfilice@ucm.es (M.F.); 4 Atrys Health, 28001 Madrid, Spain; 5 Institute of Materials Science of Barcelona (ICMAB-CSIC), Campus UAB, 08193 Bellaterra, Spain; pguardia@icmab.es; 6 ICTS—Centro Nacional de Microscopía Electrónica, Universidad Complutense de Madrid, 28040 Madrid, Spain; mitinoco@ucm.es; 7 Department of Anatomy and Embryology, Faculty of Medicine, University of Granada, 18071 Granada, Spain; beatrizgarnel@ugr.es (B.G.-P.); jcprados@ugr.es (J.P.); melguizo@ugr.es (C.M.); 8 Center of Biomedical Research (CIBM), Institute of Biopathology and Regenerative Medicine (IBIMER), University of Granada, 18100 Granada, Spain; 9 Instituto Biosanitario de Granada (ibs.GRANADA), 18014 Granada, Spain; 10 Departamento de Química Orgánica, Facultad de Ciencias, Universidad de Málaga, 29071 Malaga, Spain; jmromero@uma.es; 11 Microscopy and Dynamic Imaging Unit, Fundación Centro Nacional de Investigaciones Cardiovasculares Carlos III (CNIC F.S.P.), Calle Melchor Fernández Almagro 3, 28029 Madrid, Spain

**Keywords:** electrospraying, Ag_2_S nanoparticles, SPIONs, hybrid system, chemotherapeutic drug, drug release

## Abstract

Ag_2_S nanoparticles are near-infrared (NIR) probes providing emission in a specific spectral range (~1200 nm), and superparamagnetic iron oxide nanoparticles (SPION) are colloidal systems able to respond to an external magnetic field. A disadvantage of Ag_2_S NPs is the attenuated luminescent properties are reduced in aqueous media and human fluids. Concerning SPION, the main drawback is the generation of undesirable clusters that reduce particle stability. Here, we fabricate biocompatible hybrid nanosystems combining Ag_2_S NPs and SPION by the electrospraying technique for drug delivery purposes. These nanostructures are composed of poly(lactic-co-glycolic acid) (PLGA) as the polymeric matrix in connection with both Ag_2_S NPs and SPIONs. Initially, we fabricate a hybrid colloidal nanosystem composed of Ag_2_S NPs in connection with PLGA (PLGA@Ag_2_S) by three different routes, showing good photoluminescent (PL) properties with relatively high average decay times. Then, we incorporate SPIONs, obtaining a PLGA polymeric matrix containing both Ag_2_S NPs and SPION (PLGA@Ag_2_S@SPION). Interestingly, in this hybrid system, the location of Ag_2_S NPs and SPIONs depends on the synthesis route performed during electrospraying. After a detailed characterization, we demonstrate the encapsulation and release capabilities, obtaining the kinetic release using a model chemotherapeutic drug (maslinic acid). Finally, we perform in vitro cytotoxicity assays using drug-loaded hybrid systems against several tumor cell lines.

## 1. Introduction

Fluorescence optical imaging has emerged as a technique used in nanomedicine to obtain physiological information of organs and tissues [1]. Several fluorophores emitting in the visible (Vis, 400–750 nm) or the first near-infrared window (NIR-I, 750–900 nm) have been synthesized as emitting markers [2,3,4]. However, the main disadvantages of these types of fluorophores are: (i) low tissue penetration, (ii) reduced spatial resolution, (iii) long acquisition times, and (iv) the autofluorescence generated by living tissues [5], which generates unclear images [6]. Fluorophores emitting in the second near-infrared window (NIR-II, 1000–1400 nm) have been successfully fabricated as a luminescence alternative for in vivo applications [7,8,9]. The principal advantages of NIR-II systems are that (i) they reduce photon scattering, (ii) they provide deeper tissue penetration, and (iii) they generate a much lower autofluorescence [10]. Silver sulfide nanoparticles (Ag_2_S NPs) have recently appeared as a new type of NIR-II fluorophores [11,12], exhibiting fluorescence emission at ~1200 nm, which is an optimal emission wavelength for in vivo applications, and they have demonstrated negligible toxicity in biological organisms [13,14]. Unfortunately, Ag_2_S NPs present some drawbacks such as the reduction of the decay-time in aqueous media, which is also produced when Ag_2_S NPs are dispersed in biological media. This circumstance considerably reduces their applications in biological environments. For this reason, the fabrication of water-soluble hybrid nanosystems containing Ag_2_S NPs will avoid the undesirable fluorescence quenching, offering the possibility to be applied in biomedical applications in a higher extension. 

Superparamagnetic iron oxide nanoparticles (SPIONs) are systems in the colloidal range that improve individual properties because they are systems able to respond to an external magnetic field [15]. This circumstance is exploited in nanomedicine because it offers the possibility to be located in a specific area by the application of an external magnetic field or to promote molecular imaging by magnetic resonance techniques [16]. Indeed, it is important to mention that apart from the targeting and imaging abilities, the application of an alternating magnetic field (AMF) in the presence of SPION considerably increases their application through magnetic hyperthermia, which is used in the tumor reduction of solid tumors [17]. However, the use of pure SPION normally produces aggregates, generating unstable clusters, which reduce particle stability and results in a non-adequate alternative to be applied in nanomedicine. For this reason, their incorporation in polymeric structures, such as silica nanoparticles, microgels, or biodegradable polymers is highly desirable. 

In order to combine different nanoparticles, the fabrication of nanostructures denoted as hybrid systems has merged as a new and improved alternative in colloidal chemistry [18,19]. These nanocomposite systems are typically structured by metallic, magnetic, or semiconducting NPs in connection with an organic or inorganic polymer [20,21,22,23]. These hybrid nanomaterials have demonstrated improved applications, as they exhibit magnetic, plasmonic, or bioimaging functionalities, with the possibility to incorporate and accumulate several molecules, such as drugs, proteins, or nucleic acids, into the polymeric structure [15,22]. Several methodologies have been used for the fabrication of hybrid polymeric systems. Here we used the electrospraying technique as a simple methodology able to produce highly stable hybrid polymeric particles in the nanoscale range [24,25]. In electrospraying, when a strong electrostatic field is applied to a polymer solution held in a syringe, the pendent droplet is deformed into a Taylor cone. Then, multiple charged droplets are ejected from the tip of the Taylor cone [26,27]. An important advantage of this technique is that it can be further scaled up for mass production by using a multiplexed source spray (multi-injectors). Indeed, electrospraying includes the possibility to work by single capillarity (blend electrospraying) or through a dual-capillary (co-axial electrospraying) [28,29,30], which offers the possibility to obtain different hybrid nanostructures. Indeed, electrospraying is exploited to encapsulate different specimens, as chemotherapeutic drugs [31]; peptides [32]; DNA [33]; or antibodies into biocompatible polymers [34], such as poly(lactic-co-glycolic acid) (PLGA) [35], polylactic acid (PLA), polycaprolactone (PCL), polyvinyl alcohol (PVA), and poly(ethylene oxide) (PEO) in an easy and reproducible way [36,37,38,39]. 

For these reasons, the development of new hybrid nanostrucutres containing metallic, magnetic, or semiconducting nanoparticles, to be used in the accumulation, transport, and release of specific drugs, will supply an ideal therapeutic strategy to provide, for example, chemotherapeutic treatments more efficiently. These hybrid systems will allow to: (i) improve drug activity by the possibility of producing hyperthermia, (ii) increase drug specificity by the application of an external magnetic field, and (iii) offer an optimal fluorescence-based optical imaging. 

Here, we present the synthesis of biocompatible hybrid nanosystems by electrospraying for localized drug delivery applications. These nanostructures are composed of biocompatible PLGA in connection with Ag_2_S NPs and SPIONs (PLGA@Ag_2_S@SPION). Our methodology produces hybrid systems with PL emission at ~1200 nm (second near-infrared window), as well as magnetic responses, relevant properties for potential applications in bioimaging, and localized drug release. Our presented methodology is able to fabricate hybrid system by several routes. Thus, our methodologies include blend and co-axial electrospraying, which offer the possibility to control the location of Ag_2_S NPs and the SPIONs into the PLGA matrix. Specifically, for hybrid PLGA@Ag_2_S particles, we include three different synthetic routes, denoted as route 1, 2, and 3. For hybrid PLGA@Ag_2_S@SPION, we present two synthesis routes, denoted as route 4 and 5. We include detailed descriptions for the synthesis of hybrid particles for the five presented routes. Transmission electron microscopy (TEM), transmission electron microscopy with energy-dispersive X-ray analysis (TEM-EDX), dynamic light scattering (DLS), emission photoluminescence spectroscopy, decay time, and magnetization analysis are included as physicochemical characterization techniques. We include a stabilization study performed by TEM and DLS where we determine the polymeric degradation in the function of time. Indeed, we provide a kinetic drug release investigation by using a model chemotherapeutic drug (maslinic acid, University of Granada, Granada, Spain), a natural triterpene extracted from the coating of olives that induces apoptosis in human colon cancer cells via the mitochondrial apoptotic pathway [40]. Finally, cytotoxicity in vitro assays are also performed by using MA-loaded PLGA@Ag_2_S@SPION system and different normal or cancer cell lines, obtaining a significant inhibition of cell proliferation. Our methodology offers the possibility to create hybrid colloidal systems containing other colloidal systems, synthetic or natural polymers, or even antibody-functionalized polymers. 

## 2. Materials and Methods

### 2.1. Materials

Silver nitrate (99%), sodium diethyldithiocarbamate (NaDDTC) (ACS reagent grade), oleylamine (70%) (OLA), 1-dodecanethiol (≤98%) (DDT), O-(2-Mercaptoethyl)-O′-methylpolyethylene glycol Mw: 2000 (SH-PEG-OCH_3_), CHCl_3_ (99.6%), ethanol (99.9%), N,N-dimethylformamide (DMF, 99.9%), tetrahydrofuran (THF, 99.9%), FeCl_2_.4H_2_O, FeCl_3_.6H_2_O; oleic acid (technical grade, 90%); NH_4_OH (Ammonium hydroxide solution. ACS reagent, 28.0–30.0% NH_3_ basis), poly(D,L-lactide-co-glucolide, PLGA) M_W_ 7.000–17.000, poly(D,L-lactide-co-glycolide)(50:50)-b-poly(ethylene glycol, PLGA-PEG) Mw 10.000–2.000, and phosphate-buffered saline (PBS) tablets were purchased from Sigma-Aldrich and used as received. Dulbecco’s Modified Eagle’s Medium (DMEM) (Sigma-Aldrich, Madrid, Spain). Fetal bovine serum (FBS) (Gibco, Madrid, Spain). DMEM-HAM-F12. Horse serum. Antibiotics: EGF (epidermal growth factor), hydrocortisone, choleric toxin and Insulin-Transferrin-Selenium. DMEM/F12 was purchased from Lonza (Basel, Switzerland). DMEM, High glucose, GlutaMAX Supplement (DMEM+GlutaMAX) was purchased from Gibco (Waltham, MA, USA). Minimum Essential Medium Eagle (MEM) was purchased from Merck (Darmstadt, Germany). Non-essential amino acids (NEAA) and sodium pyruvate were obtained from Hyclone (Logan, UT, USA), while penicillin/streptomycin from Lonza (Basel, Switzerland). Dimethylthiazolyl-diphenyl-tetrazolium bromide (MTT), and dimethyl sulfoxide (DMSO) were bought by Merck (Darmstadt, Germany).

### 2.2. Characterization

Transmission electron microscopy (TEM) investigations were performed using a JEOL JEM 1400 (JEOL, Tokyo, Japan) microscope working at 80 kV. High-angle annular dark field (HAADF) scanning TEM and energy dispersive X-ray analysis (EDX) mappings were conducted using a FEI Talos F200X (FEI, Hilssboro, OR, USA) equipped with an EDX detector. Sample preparation was carried out by placing 10 μL of each sample on a carbon-coated TEM grid. Scanning electron microscopy (SEM) was performed by using a scanning electron microscope (SEM, JEOL JSM 6335F, Tokyo, Japan) working at 15 KV. Samples were sputtered with gold during 90 sec to minimize charging effect by using a Quorum Q150RS sputtering. Dynamic light scattering (DLS) measurements were performed on a Zetasizer Nano S (Malvern Instruments, Malvern, UK) using a detection angle of 173°. The Nano S used a 4 mW He–Ne laser operating at a wavelength of 633 nm. The intensity-averaged particle diameter and the polydispersity index values (an estimate of the distribution width) were calculated from the cumulant analysis. High-performance liquid chromatography (HPLC) Agilent 1260 Infinity II with a C18 column Agilent InfinityLab Poroshell 120 (Agilent, Santa Clara, CA, USA) was used for the determination of maslinic acid in the in vitro control release. The emission spectra upon illuminating the samples with an 800 nm CW laser were collected with an Andor iDus InGaAs 491 cooled to −90 °C. Luminescence was detected by a liquid-nitrogen-cooled NIR photomultiplier tube (Hamamatsu, R550-72, Shizuoka, Japan). Magnetic properties were measured using a Superconducting Quantum Interference Device (SQUID, MPMS5XL magnetometer, Quantum Design, San Diego, CA, USA). Hysteresis loops at 5 and 300 K under a maximum field of ±60 kOe were measured. Magnetization values were normalized by the total amount of sample (magnetic and non-magnetic).

### 2.3. Synthesis of Ag_2_S@DDT and Ag_2_S@SH-PEG-OCH_3_ NPs

Previously to the synthesis of Ag_2_S@DDT NPs, the precursor silver diethyldithiocarbamate, Ag(DDTC), was obtained by the previously reported method described by Yaping and col. Briefly, in two Erlenmeyer flasks, an equimolar amount of AgNO_3_ (4.25 g, 0.05 mol) and Na(DDTC) (5.63 g, 0.05 mol) were dissolved in H_2_O (200 mL each), under sonication and magnetic stirring. After that, the AgNO_3_ solution was added drop by drop to the Na(DDTC) solution by using an addition funnel. The reaction was performed at room temperature in the absence of light, and it was left to react for 12 h under vigorous magnetic stirring. Then, the yellow precipitate was filtered using a Büchner funnel, and it was washed with H_2_O three times. Finally, the obtained solid was dried for 4 h at 60 °C under vacuum, and it was stored and protected from the light and humidity.

The synthesis of Ag_2_S@DDT NPs was performed by following the same procedure previously reported by us [13]. Basically, a given amount of Ag(DDTC) (25 mg, 0.1 mmol) was added into a two-neck round-bottom flask at room temperature. Then, 5 mL of a solvent mixture containing OLA and DDT (50:50) was added. Once the Ag(DDT) was dissolved, the mixture was placed under vacuum for 10 min to remove air, and then, it was filled with N_2_. After that, the mixture was heated to 180 °C under slow magnetic stirring. The reaction was kept for 1 h and then left to cool down to room temperature. After that, the synthesized NPs were collected by adding 10 mL of ethanol to the raw product and by centrifugation at 10,062 rcf for 10 min. This cleaning process was repeated twice. Finally, the as-prepared product was dispersed in 10 mL of chloroform and stored for further steps.

Before introducing Ag_2_S NPs into the electrospraying device, it is necessary for a previous ligand exchange step due to DDT not being stable in the solvent mixture of DMF:THF (30:70).

An excess of the new ligand SH-PEG-OCH_3_ was added to Ag_2_S NPs, and the mixture was sonicated for 5 min. After that, the Ag_2_S@SH-PEG-OCH_3_ NPs were washed with EtOH at least two times. Precipitation step was carried out adding a five times excess of hexane and centrifugation at 3622 rcf for 4 min. Finally, the pellet was redispersed in a mixture of DMF:THF (30:70) to reach a final concentration of 1 mg/mL.

### 2.4. Fabrication of Nanoparticles by Electrospraying Technique

As was previously mentioned, we used two methodologies in the fabrication of these hybrid nanoparticles, single-capillary and dual-capillarity, also named blend and co-axial electrospraying, respectively. In both cases, the positive electrode was connected to the injector and the negative electrode was connected to the collector placed, which was located 15 cm away from the tip of the needle.

**Scheme 1:**
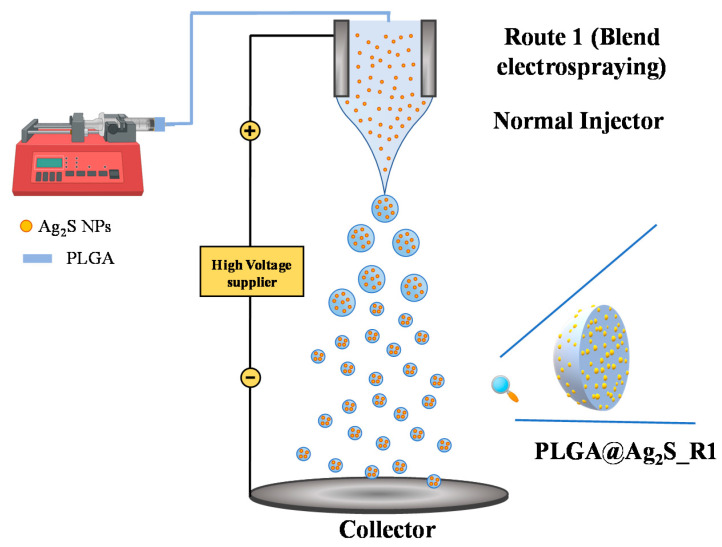
Schematic representation for the synthesis of PLGA@Ag_2_S_R1 NPs.

In blend conformation, the dispersion was sprayed using a syringe pump through a metal needle (outer diameter 0.9 mm and inner diameter 0.6 mm) at a flow rate of 1 mL/h, through a PTFE capillary (outer diameter 1.6 mm, inner diameter 0.8 mm) and at an applied voltage of 8 kV. Scheme 1 illustrates the fabrication by blend electrospraying for the denoted PLGA@Ag_2_S_R1 system.

**Scheme 2:**
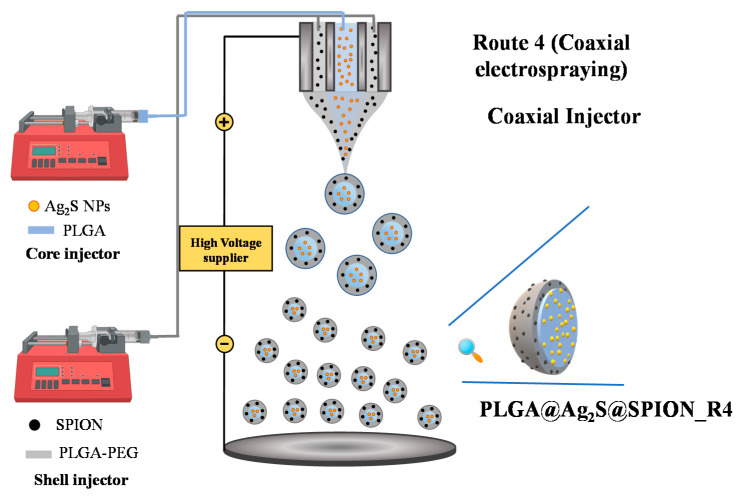
Schematic representation for the synthesis of PLGA@Ag_2_S@SPION_R4.

Co-axial electrospraying is developed by using an external capillary (outer diameter of 1.7 mm and an inner diameter of 1.4 mm) and an internal capillary (outer diameter of 0.9 mm and an inner diameter of 0.6 mm). The outer flow rate was 1 mL/h while the inner flow was 0.5 mL/h. This increase in the volume sprayed leads to an increase in the applied voltage until 12.5 kV. Scheme 2 illustrates the fabrication by co-axial electrospraying for the here denoted PLGA@Ag_2_S@SPION_R4 system

Each hybrid system has a matrix obtained from 1% PLGA and 0.1% of PLGA-PEG in mass from the total solvent mixture DMF:THF (30:70). For R1 and R1@Drug routes, both polymers are injected through the same syringe pump. However, in R2, R3, R4, and R5 routes, PLGA was injected through the inner flow and PLGA-PEG in the outer flow.

The differences between electrospray hybrid systems are mainly due to the position of the active elements (Ag_2_S@SH-PEG-OCH_3_, SPION, and Maslinic acid). The denoted name used for a hybrid system, the specific location, as well as the percentages of nanoparticles or drug used during synthesis are shown in Table 1. Indeed, Schemes S1–S3 include a schematic representation for the synthesis of PLGA@Ag_2_S_R2, PLGA@Ag_2_S_R3, and PLGA@Ag_2_S@SPION_R5 hybrid systems.

### 2.5. Synthesis of Superparamagnetic Iron Oxide Nanoparticles (SPION)

The synthesis of SPIONs was performed by following a modification of the previously reported co-precipitation method (Massart’s method) [41,42]. In specific, in a 100 mL three-neck round bottom flask, 30 mL of MilliQ water was introduced and purged with N_2_ during 10 min. Then, under magnetic stirring, the mixture of Fe^2+^/Fe^3+^ salts composed of 2 g of FeCl_2_∙4H_2_O and 4.8 g of FeCl_3_∙6H_2_O was added. The mixture was heated to 90 °C with 0.85 mL of oleic acid. After reaching this temperature, a mixture composed of 10 mL of NH_4_OH and 10 mL of MilliQ water was added dropwise, resulting in a color change from orange to black. The reaction was maintained at these conditions for 2.5 h. After this time, the heat was removed and the reaction was left to cool down at room temperature. Finally, the generated SPIONs were recovered and purified by magnetic decantation and washed several times with a mixture of DMF:THF (30:70).

### 2.6. Encapsulation Efficiency

The efficiency of maslinic acid incorporated after PLGA@Ag_2_S@maslinic acid preparation was calculated by determining the amount of maslinic acid (MA) in chloroform extracts of known weights of dried hybrid systems. This value was then subtracted from the amount of MA used to prepare the PLGA@Ag_2_S@maslinic acid sample.

The encapsulation efficiency (*EE*), expressed as a percentage, was calculated according to Equation (1).

(1) $$EE\left(\%\right)=\frac{W_{initial\;drug}-W_{free\;drug}}{W_{initial\;drug}}\times 100$$

The amount of MA in the samples was measured by HPLC analysis (see Section 2.2 for details). The mobile phase was acetonitrile:water (70:30, *v*/*v*), at a flow rate of 1 mL·min^–1^, with detection at 227 nm. A calibration curve was accordingly prepared. To determine the amount of drug incorporated into the PLGA@Ag_2_S@maslinic acid, 3 mL was centrifuged (8000 rpm) for 1 h. The aqueous layer was decanted and then extracted with chloroform (3 × 3 mL). The chloroform extracts were dried over MgSO_4_ and concentrated to dryness. The residue was reconstituted in 1 mL water:acetonitrile (70:30, *v*/*v*) and injected into the HPLC system to determine the amount of MA.

### 2.7. Cell Culture and In Vitro Cytotoxicity Assays

The A549 human lung adenocarcinoma cell line, MCF-7 human breast cancer cell line, and T-84 human colon cancer cell line were purchased from the American Type Culture Collection (Rockville, MD, USA). The MCF10A breast cell lines were obtained by the Instrumentation Service Center (CIC, University of Granada, Granada, Spain).

For in vitro cytotoxicity assays, cells were seeded at densities of 5 × 10^3^ cells/well (A549, MCF-7, T-84) in 48-well plates with DMEM supplemented with 10% fetal bovine serum (FBS) and a 1% antibiotic mixture. MCF10A cells were seeded at densities of 10 × 10^3^ cells/well in 48-well plates with DMEM-HAM-F12 supplemented with 5% horse serum, 1% antibiotic mixture, EGF (20 µg/mL), hydrocortisone (0.5 µg/mL), choleric toxin (100 ng/mL), and insulin–transferrin–selenium (10 µg/mL). All the cell lines were incubated overnight at 37 °C, in a 5% CO_2_ humidified atmosphere, and treated with increasing concentrations (0.1–100 µg/mL) of nanoparticles (PLGA@Ag_2_S_R1, PLGA@Ag_2_S_R2, and PLGA@Ag_2_S@SPION_R4) for 72 h. 

A sulforhodamine B (SRB) assay was carried out following our protocol [43]. Briefly, the cells were fixed with trichloroacetic acid at 10%, stained with Sulforhodamine B, and the dye was lifted with Trizma^®^ (Sigma-Aldrich, Madrid, Spain) at 10% in a new 96-well plate. Finally, the optical density (OD) at 492 nm was measured in a spectrophotometer EX-Thermo Multiskan (Flow, Irvine, CA, USA). Cell survival (%) was calculated according to the following equation:

$$Proliferation\;\left(\%\right)=\frac{Treated\;cells\;OD-blank}{Control\;OD-blank}\times 100$$

### 2.8. Cell Culture and MTT Assay

MDA-MB-231, Hek 293T, Hep G2, and MEF cell lines were used to perform cell viability assay. MDA-MB-231 cells were grown and maintained in DMEM/F12 supplemented with 10% FBS, 1% NEAA, 1% sodium pyruvate 100 mM, and 1% penicillin/streptomycin. Hek-293T cells were grown and maintained in DMEM+GlutaMAX supplemented with 10% FBS, 1% sodium pyruvate 100 mM, and 1% penicillin/streptomycin. Hep G2 cells were grown and maintained in MEM supplemented with 10% FBS, 1% NEAA, and 1% glutamine. MEF cells were grown and maintained in DMEM supplemented with 10% FBS, 1% sodium pyruvate 100 mM, and 1% penicillin/streptomycin. These cultures were maintained at 37 °C and in an atmosphere with 5% CO_2_.

For in vitro cytotoxicity assay (MTT) with each cell line, cells were seeded at densities of 7.5 × 10^3^ cells/well (MDA-MB-231, Hek 293T and Hep G2) or at 5 × 10^3^ cells/well (MEF) with its corresponding medium in a 96-well culture plate and grown for 48 h at 37 °C. Then, cells were incubated for 24 h at 37 °C with increasing concentrations (0.1–100 µg/mL) of nanoparticles redispersed in culture media without phenol red (PLGA@Ag_2_S_R1 and PLGA@Ag_2_S@maslinic acid). After that, all the solutions were discarded and replaced by 100 µL of medium (without FBS and phenol red) and 10 µL of a 12 mM MTT solution in medium in each well. The reaction was incubated for 3 h, and then 85 µL of supernatants were removed, and 100 µL of DMSO were added to dissolve the formazan, leaving it for 15 min in gentle agitation. Cell viability was estimated by measuring absorbance at 570 nm, knowing that the higher the absorbance, the higher the viability.

### 2.9. In Vitro Drug Control Release

The kinetic release was evaluated using the previous fabricated PLGA@Ag_2_S@maslinic acid. For this aim, 0.5 mL of a drug-loaded nanoparticles dispersion at 2 mg/mL in PBS (pH 7.4) was placed on a Transwell^®^ (Corning Incorporated, Kennebunk, ME, USA) permeable support with 0.4 µm of polycarbonate membrane (*n* = 3). The well was filled with 1.5 mL of PBS (pH 7.4), and then, the suspension was incubated at 37 °C under gentle stirring of 150 rpm for 24 h. For aliquot collecting, the solution outside of the Transwell^®^ insert was replaced with the same volume (1.5 mL) of fresh PBS. The amount of maslinic acid in each aliquot was determined by HPLC. The experiment was repeated in triplicate. Excel for Mac 2020 with the Solver tool was used for the calculation of the release rate constants and the determination of the correlation coefficients (R^2^).

### 2.10. Statistical Analysis

All results are expressed as the mean ± standard deviation of three replicates. In all in vitro experiments the groups were compared by one-way analysis of variance (ANOVA). A probability value (*p*) of less than 0.05 was considered to be statistically significant.

## 3. Results and Discussions

### 3.1. Synthesis of Ag_2_S@DDT and Ag_2_S@SH-PEG-OCH_3_ Nanoparticles

**Figure 1:**
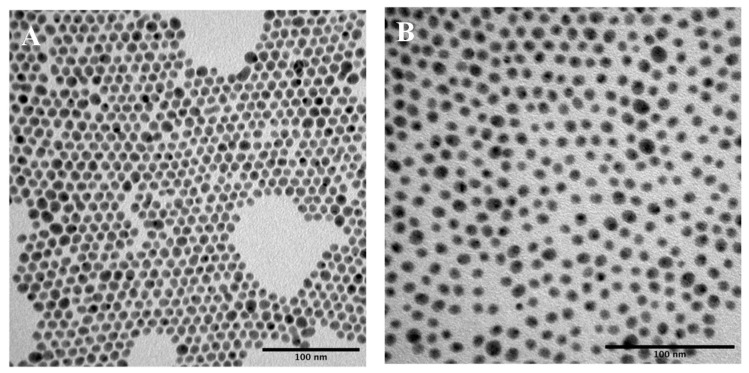
Representative TEM images for (**A**) Ag_2_S@DDT and (**B**) Ag_2_S@SH-PEG-OCH_3_ NPs.

Initially, Ag_2_S NPs were synthesized in CHCl_3_ using 1-dodecanethiol (DDT) as a colloidal stabilizer ligand (Ag_2_S@DDT) [18]. This method produces a highly dispersible colloidal system, but is only stable in organic solvents. To obtain NPs able to be dispersed in biological media, the Ag_2_S@DDT NPs were functionalized with SH-PEG-OCH_3_ by ligand exchange process, removing the DDT moieties and exchanging them with the PEG units (see Material and methods section for details). This process resulted in stable colloidal dispersion in both organic and aqueous solutions. Figure 1 includes representative TEM images of Ag_2_S@DDT and Ag_2_S@SH-PEG-OCH_3_ NPs. As observed, well-distributed and monodisperse Ag_2_S NPs were obtained in both cases. The average particle size measured from TEM images was 10.2 ± 0.9 nm and 10.6 ± 0.9 for Ag_2_S@DDT and Ag_2_S@SH-PEG-OCH_3_ QDs, respectively. The particle distribution of 100 particles is included in Figure S1, these histograms show a narrow distribution in both cases, which indicates a high monodispersity of particles. DLS measurements for Ag_2_S@DDT and Ag_2_S@SH-PEG-OCH_3_ both in CHCl_3_ resulted in a hydrodynamic diameter (D_h_) of 10.4 ± 1.8 and 16.4 ± 2.1 nm, respectively. This increase in the hydrodynamic diameter is due to the presence of the ethylene glycol units on the Ag_2_S surface after ligand exchange. Interestingly, a more detailed analysis from TEM images shows that a higher inter-particle distance was observed for Ag_2_S@SH-PEG-OCH_3_ compared with pure Ag_2_S@DDT NPs. This is due to the presence of ethylene glycol units on the Ag_2_S NP surface. Figure S2 shows the photoluminescence (PL) emission spectra for Ag_2_S@DDT (black line) and Ag_2_S@SH-PEG-OCH_3_ (red line) NPs, both in CHCl_3_, obtained under optical excitation of 800 nm. In both cases, an emission band located at ~1200 nm was produced. Interestingly, the PL intensity for both colloidal shows no significant differences. Figure S3 shows optical images of colloidal dispersion for Ag_2_S@DDT and Ag_2_S@SH-PEG-OCH_3_ in water at a concentration of 1 mg/mL. As is observed, the aqueous dispersion of Ag_2_S@DDT is turbid, and it precipitates after 12 h. However, the aqueous colloidal dispersion containing Ag_2_S@SH-PEG-OCH_3_ NPs showed a clear solution that does no precipitate in several weeks. Due to this higher stability of Ag_2_S@SH-PEG-OCH_3_ NPs compared with Ag_2_S@DDT, we further used the PEGylated system for the synthesis of the hybrid system.

### 3.2. Synthesis of Ag_2_S@PLGA Nanoparticles

**Figure 2:**
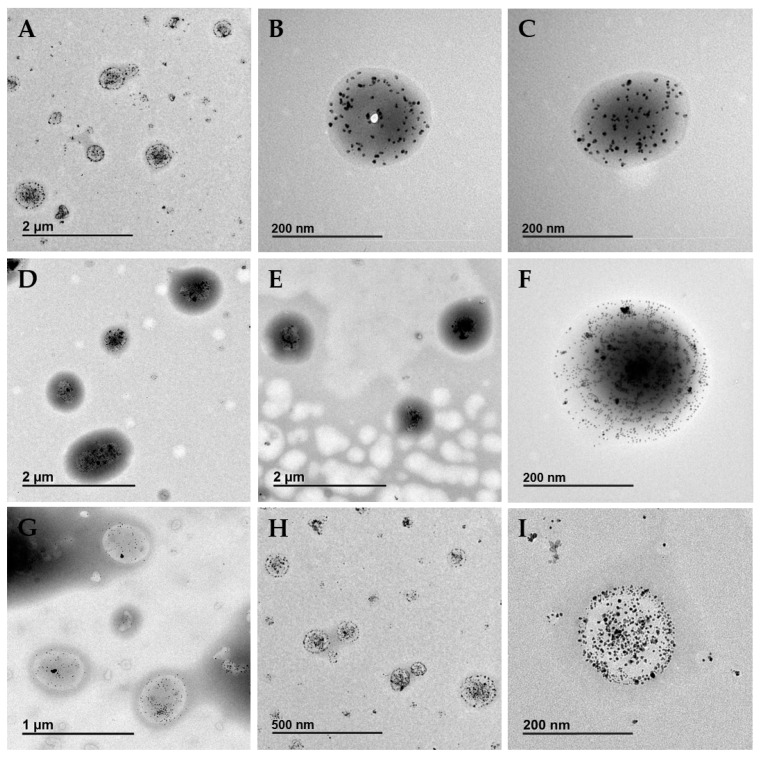
Representative TEM images for (**A**–**C**) PLGA@Ag_2_S_R1 NPs obtained by route 1. (**D**–**F**) PLGA@Ag_2_S_R2 NPs obtained by route 2, and (**G**–**I**) PLGA@Ag_2_S_R3 NPs obtained by route 3.

As mentioned, we present three different synthetic routes to fabricate PLGA@Ag_2_S NPs by electrospraying process (see Section 2 for details). By route 1, colloidal dispersion of Ag_2_S@SH-PEG-OCH_3_ NPs was mixed with a PLGA/PLGA-PEG solution, and this mixture was subjected to blend electrospraying. This addition of a small amount of PLGA-PEG was performed in order to obtain a particle surface functionalized with PEG moieties. This functionalization is essential for future bio-applications since it will confer colloidal stability to the system and can also abolish opsonization processes, and consequently, a fast elimination by the mononuclear phagocytic system (MPS). Figure 2A–C include representative TEM images at different magnifications for PLGA@Ag_2_S_R1 NPs, generated by route 1. As observed, the obtained hybrid colloidal system was structured by well-distributed spherical Ag_2_S NPs in connection with a PLGA matrix. A detailed study of TEM and DLS in the function of time will be presented below. Concerning route 2, co-axial electrospraying was used for the generation of the denoted hybrid PLGA@Ag_2_S_R2 NPs system (see Section 2 for details). By using this synthesis route, the inner dispersion was composed of a mixture of Ag_2_S@SH-PEG-OCH_3_ NPs and PLGA, and the external injector contained a PLGA-PEG solution. Figure 2D–F show representative TEM images for PLGA@Ag_2_S_R2 NPs generated by route 2 at different magnifications. As observed, the fabricated hybrid system exhibits a hybrid structure formed by polymeric PLGA NPs decorated with Ag_2_S NPs. In route 3, co-axial electrospraying was also performed for the generation of the hybrid PLGA@Ag_2_S NPs; however, in this case, the Ag_2_S@SH-PEG-OCH_3_ dispersion was located in the external injector with the PLGA-PEG solution, and the PLGA was introduced in the inner injector (see Section 2 for details). TEM images of PLGA@Ag_2_S_R3 NPs at different magnification obtained by route 3 are included in Figure 2G–I. Again a hybrid structure formed by a polymeric matrix containing well-dispersed Ag_2_S NPs is observed. A detailed observation of TEM images show a great number of Ag_2_S NPs situated on the external PLGA surface. This circumstance will be further demonstrated by the EDX elemental analysis. Figure S4 includes SEM images for PLGA@Ag_2_S_R1 NPs

**Figure 3:**
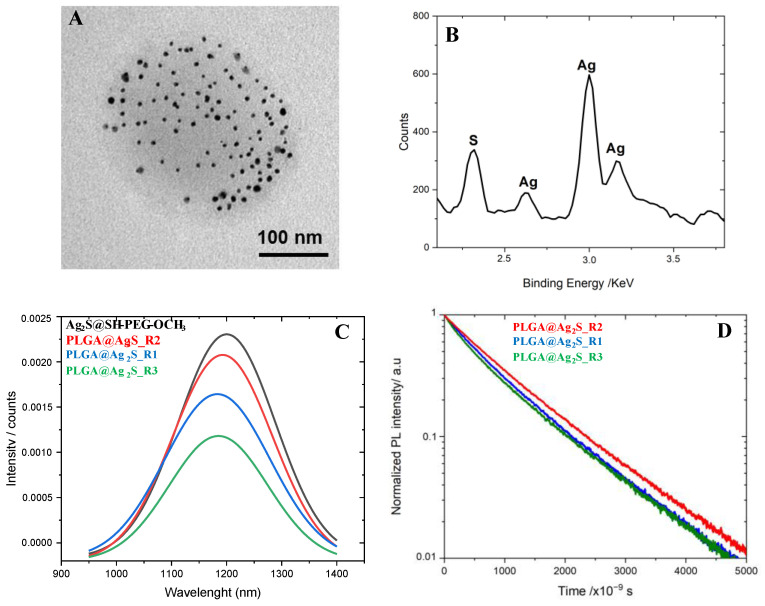
(**A**,**B**) Energy-dispersive X-ray spectroscopy spectra of the included PLGA@Ag_2_S NPS showing the Ag and S regions. (**C**) PL emission spectra and (**D**) PL decay curves for Ag_2_S@SH-PEG-OCH_3_ NPs (black line), PLGA@Ag_2_S_R1 (blue line), PLGA@Ag_2_S_R2 (red line), and PLGA@Ag_2_S_R3 green line.

Further evidence of the presence of Ag_2_S NPs into the PLGA structure was achieved by TEM-EDX analysis. Figure 3A,B include TEM image and energy dispersive X-ray (EDX) spectrum for PLGA@Ag_2_S NPs fabricated by route 1 (PLGA@Ag_2_S_R1 NP). EDX quantification of the spectrum shown in Figure 3B was performed, obtaining 67 at %Ag Ag:S ratio, which is, indeed, a very similar result to the expected value for a stoichiometric Ag_2_S nanoparticle. A mean Ag:S ratio composition of 65.5 ± 1.5% Ag and 34.5 ± 1.3% S was obtained after averaging several EDX quantification results collected from different areas among the sample. In that regard, Figure S5 includes four EDX spectra for PLGA@Ag_2_S acquired in different areas. The relatively small measured standard deviation confirms the compositional homogeneity of the Ag_2_S nanoparticles in study. The compositional spectrum shows both the Ag and S regions in all cases. The chemical composition entails an Ag:S ratio of 2:1, in concordance with the expected 2:1 (66% Ag, 33% S) [13].

**Figure 4:**
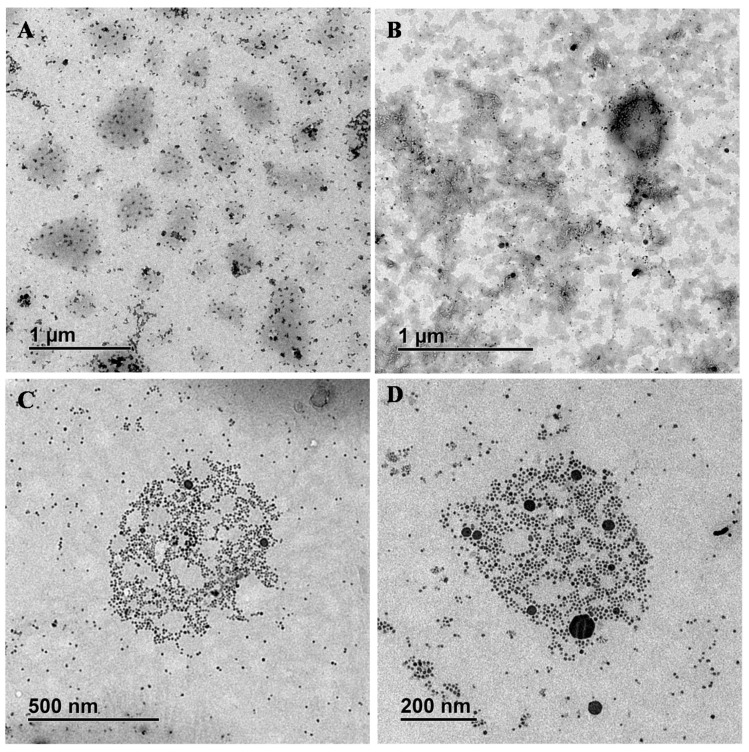
TEM images for hybrid PLGA@Ag_2_S_R1 particles performed at (**A**) 0 h, (**B**) 8 h, (**C**) 24 h, and (**D**) 48 h.

Figure 3C includes the photoluminescence (PL) emission spectra for Ag_2_S@SH-PEG NPs (black line) as well as the three fabricated hybrid PLGA@Ag_2_S systems dispersed in a buffer solution. The Ag_2_S NP concentration was adjusted to 0.15 mg/mL in all cases. The PL emission spectrum for Ag_2_S@DDT nanoparticles is not included, as this colloidal sample cannot be dispersed in an aqueous buffer solution (see Figure S3). As is observed, all samples exhibit a well-defined emission peak at ~1200 nm. The highest PL intensity was produced by the Ag_2_S@SH-PEG-OCH_3_ sample, but the PL emission intensity exhibited by the three hybrid PLGA@Ag_2_S systems is in the same order compared with the Ag_2_S@SH-PEH-OCH_3_ system. Specifically, the highest PL intensity was supplied by the PLGA@Ag_2_S_R2 sample. In this system, the coaxial electrospraying enables the Ag_2_S NPs to be located in the core of the PLGA matrix (see Scheme 2 and Figure 3). The second PL emission intensity was provided by the PLGA@Ag_2_S_R1 hybrid system. By this route, blend electrospraying is used to obtain the colloidal hybrid system, and, as Scheme 1 and Figure 2 reveal, the Ag_2_S NPs are homogeneously distributed for the entire PLGA matrix. Finally, the lowest PL emission intensity was obtained by particles fabricated by route 3 (PLGA@Ag_2_S hybrid system), where coaxial electrospraying was also used to obtain the hybrid system, and the Ag_2_S NPs were on the external part of the PLGA matrix (see Scheme S2 and Figure 4). These results are in concordance with the fact the PL emission intensity of Ag_2_S NPs is quenched by the presence of water molecules. As is observed, the sample with the highest PL intensity is provided by the PLGA@Ag_2_S_R2 system, where the Ag_2_S are mainly located into the PLGA matrix, not in proximity with water molecules. Then, the hybrid particles are obtained by route 1, where Ag_2_S NPs are homogeneously distributed through the entire PLGA matrix. Finally, the sample with the lowest PL intensity is produced by the PLGA@Ag_2_S_R3, where Ag_2_S are principally located at the periphery of the PLGA matrix, which are more in contact with water molecules. Figure 3D includes luminescence decay times for the hybrid particles fabricated by the three different routes. After the exponential analysis, results fit with a biexponential curve, obtaining decay time values of t_1_ = 1311 ns and t_2_ = 687 nm for route 2, t_1_ = 1107 nm and t_2_ = 379.8 nm for route 1, and t_1_ = 480.5 nm and t_2_ = 1122 ns 994 ns route 3, which fits with the previously described PL emission results. Figure S6 includes the analyzed lifetime profiles where the mentioned exponential fits are presented.

### 3.3. Stability Assay

In order to determine the degradation behavior, and thus, the colloidal stability of the fabricated hybrid systems, we performed a stabilization assay for the three different synthesized hybrid systems (PLGA@Ag_2_S_R1, PLGA@Ag_2_S_R2, and PLGA@Ag_2_S_R3). The stability assay involving these colloidal systems was developed by dispersion of 10 mg of each sample in 10 mL of a PBS buffer solutions, at 37.5 °C and under gentle mechanical shaking (3500 rpm). Then, samples for DLS and TEM analysis were collected at different times (0, 1, 2, 5, 8, 24, and 48 h). Figure 4 and Figure S7 include TEM images and DLS measurements for the hybrid PLGA@Ag_2_S_R1 system performed several times. As observed, during the first 2–3 h the integrity of the PLGA nanostructure remains intact. Consequently, this first stage guarantees the colloidal stability of hybrid systems, thus obtaining a majority population of dispersed particles centered around 140–164 nm. In a second stage, between 5 and 8 h of particle dispersion, the hybrid system begins to both aggregate and degrade, as is observed in Figure 4B and Figure S7B. Thus, the polymer begins to join, resulting in larger particles, or clusters, obtaining centered populations from two main sizes, 342 nm, and 2–3 µm. Between 24 and 48 h, Figure 4C,D, respectively, TEM images show both a degraded polymer and some areas with free Ag_2_S NPs. At this state, DLS measurements show a particle size of about 190–122 nm. The decrease in the particle size is produced because clusters sediment at the bottom of the vial and condense, forming a bulky structure, resulting in aggregated Ag_2_S NPs or degraded residual polymer.

The PLGA@Ag_2_S_R2 hybrid system shows the same behavior in terms of colloidal stability. Figures S8 and S9 include TEM images and DLS measurements for the stability analysis performed with the hybrid system prepared at route 2. Again, during the first 0–2 h, we observed good integrity of the PLGA nanostructure, Figure S9A, showing colloidal stability with particle size between 142–190 nm. In a second stage, between 5 and 8 h of particle dispersion, the same aggregation and degradation effect was observed, as shown in Figures S8B and S9B. Thus, obtaining centered populations from two main sizes, 298 nm and 2 µm. Finally, data at 24 and 48 h, Figure S8C,D, respectively, show TEM images including both a degraded polymer and some areas with free Ag_2_S NPs. At this state, DLS measurements show particle size of about 106–160 nm. The decrease in the particles size is produced because polymeric clusters again sediment at the bottom of the vial and condense, forming a bulky structure, resulting in aggregated Ag_2_S NPs or residual degraded polymer.

Concerning the hybrid system fabricated by route 3 (PLGA@Ag_2_S_R3), particles present a slightly better stabilization under the same incubation conditions, probably due to the presence of a higher number of Ag_2_S on the PLGA surface. In the first hours, it is observed how the dispersed sample containing these nanoparticles is stable for at least 4 h in a population centered between 164 and 190 nm (Figure S10A, DLS data do not show). In the second stage, it seems that the two previously defined effects coexist, degradation and aggregation (Figure S10B). This fact causes two very different populations to coexist in dissolution. One effect is the degradation of the PLGA nanoparticles between 5 and 8 h, and on the other hand, the formation of aggregated particles or clusters (as in the other hybrid systems R1 and R2). After 24 h, the clusters sediment to the bottom of the vial, and only the small Ag_2_S NPs and the rest of the degraded polymer nanoparticles remain in suspension (Figure S10C,D). 

### 3.4. Synthesis of Hybrid Ag_2_S@PLGA@SPION Nanoparticles

**Figure 5:**
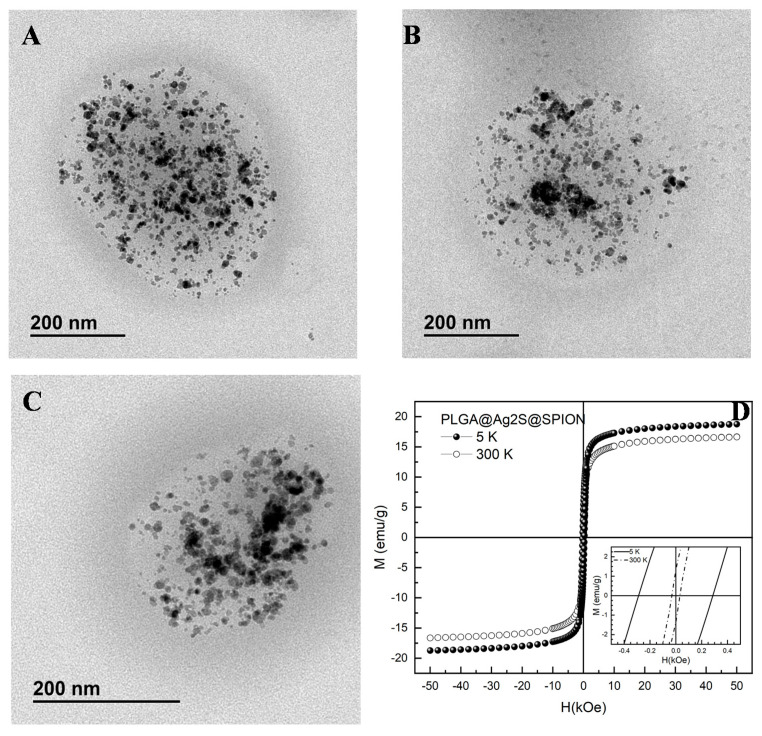
Representative TEM images for the hybrid PLGA@Ag_2_S@SPION_R4 system. (**A**–**C**). Magnetization as a function of the applied field at 5 and 300 K (filled and empty squares respectively) for PLGA@Ag_2_S@SPION_R4. Inset represents the low field region to evaluate the coercive fields at 5 and 300 K (straight and dashed lines respectively) (**D**).

After the synthesis and characterization of PLGA@Ag_2_S NPs, we include in our investigations the fabrication of a hybrid colloidal system containing more than one type of nanoparticle. We successfully achieve the fabrication of hybrid colloidal systems containing Ag_2_S NPs and SPIONs, denoted as PLGA@Ag_2_S@SPION NPs. As was detailed in the materials and methods section, magnetic NPs were fabricated by the coprecipitation method and resulted in a SPION of 18.2 ± 2.1 nm. For the synthesis of these hybrid systems, we selected the denoted route 4 and route 5, both by co-axial electrospraying (see material and methods section for details) because they offer the possibility to selectively incorporate nanoparticles in the outer or inner part of the coaxial injector. Figure 5 includes TEM images for the resulting PLGA@Ag_2_S@SPION_R4 NPs and the magnetic characterization of those. The magnetic characterization of PLGA@Ag_2_S@SPION_R4 NPs, Figure 5D, confirms a superparamagnetic behavior at room temperature of the hybrid system, with a ferromagnetic one at 5 K (Figure 5D). The saturation magnetization values measured at 5 and 300 K were 18 and 15 emu/g, respectively, which are below the ones measured for the SPIONs, see Figure S11. That deviation results from providing the saturation magnetization values (emu/g) from the whole system (i.e., PLGA@Ag_2_S@SPION_R4 and SPIONs respectively). Remarkable, although there is a presence of diamagnetic materials in PLGA@Ag_2_S@SPION_R4 samples (i.e., PLGA and Ag_2_S). Finally, the hybrid system showed a clear ferromagnetic and superparamagnetic behavior at 5 and 300 K, respectively, with saturation magnetization values appealing for their use in biomedical applications.

**Figure 6:**
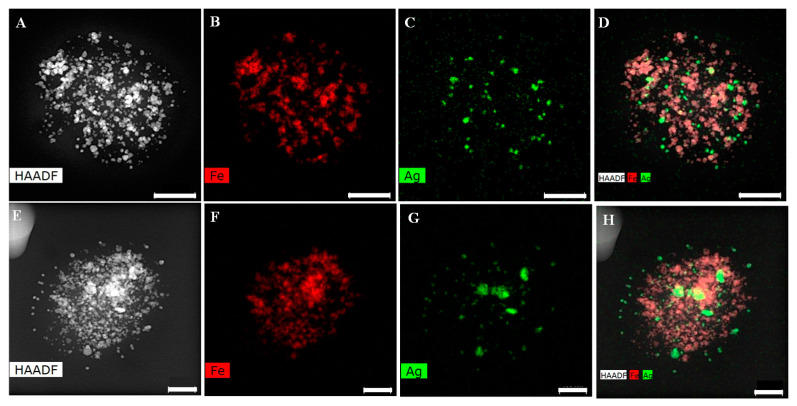
(**A**,**E**) HAADF-TEM image for hybrid PLGA@Ag_2_S@SPION_R4 and PLGA@Ag_2_S@SPION_R5 NPs, (**B**,**C**) EDX elemental mapping analysis of iron and silver for hybrid PLGA@Ag_2_S@SPION_R4 NPs. (**F**,**G**) EDX elemental mapping analysis of iron and silver for hybrid PLGA@Ag_2_S@SPION_R5 NPs. (**D**,**H**) EDX elemental mapping of mixed iron and silver for the two hybrid colloidal systems. Scale bar is 100 nm.

We also studied the particle stability for hybrid systems once they were deposited on the TEM grid, to know the particle durability. Figure S12A,B includes representative TEM images for hybrid PLGA@Ag_2_S@SPION_R4 particles freshly prepared and after 6 weeks deposited on the TEM grid, respectively. Figure S12C,D includes TEM images for PLGA@SPION@Ag_2_S_R5 freshly prepared and after 6 weeks deposited on the TEM grid, respectively. As is observed, after this period, particle degradation is not appreciated in any case, which suggests high particle durability when the hybrid system is in a dry state. A more appropriate characterization concerning composition and particle location for the hybrid PLGA@Ag_2_S@SPION system was achieved by HAADF-TEM and EDX elemental mapping analysis, which were included in Figure 6. For hybrid PLGA@Ag_2_S@SPION_R4 NPs (Figure 6A–D), a plethora of Ag_2_S NPs and SPIONs can be distinguished in the PLGA matrix, thus supporting the presence of both colloidal systems. It is important to note that by this coaxial route of synthesis, the Ag_2_S NPs are situated in the internal injector and the SPIONs in the outer one. The EDX spectrum extracted from the TEM images of Figure 6A showed a percentage of Ag and Fe of 39.4% and 60.6%, respectively. Figure 6E–I includes HAADF-TEM and EDX elemental mapping for hybrid PLGA@Ag_2_S@SPION_R5 NPs. Again, it is observed that the PLGA matrix contains both types of nanoparticles. By route 5, the colloidal dispersion containing Ag_2_S NPs was introduced into the external injector and the SPIONs in the internal one. A more detailed observation of Figure 6D,I, which corresponds to the EDX elemental mapping for Ag and Fe for PLGA@Ag_2_S@SPION_R4 and PLGA@Ag_2_S@SPION_R3 NPs, respectively, showed a different distribution of Ag_2_S NPs and SPIONs in each case. For route 4 (Figure 6D), the Ag_2_S NPs (green dots) are mainly located inside the PLGA matrix and the SPIONs (red dots and gray arrows) outside the PLGA matrix. On the contrary, for hybrid particles fabricated by route 5, the Ag_2_S NPs (green dots and white arrow) are situated outside the PLGA matrix, and the SPION (red dots) inside the PLGA matrix. These results are in concordance with the distribution of NPs established in the co-axial injector during the electrospraying process (see Material and Methods for details). 

These important results demonstrate that the particle location can be modified in the PLGA matrix by introducing the appropriate NP dispersion in the proper injector during the co-axial electrospraying process. As far as we are concerned, this is the first reported work concerning hybrid systems containing Ag_2_S and SPIONs where the location of both systems can be controlled during the synthesis process.

### 3.5. Cytotoxicity Assays

**Figure 7:**
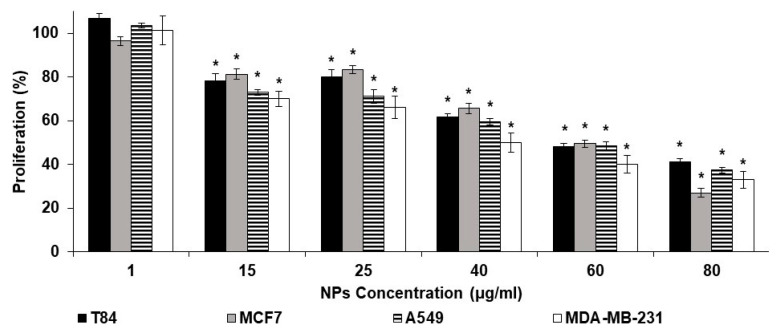
Cell proliferation assay of A549, MCF7, MDA-MB-231, and T84 tumor cell lines treated with PLGA@Ag_2_S_R1, including maslinic acid. The percentages of proliferation of the cell lines obtained are shown. Data represent the mean values ± SD of triplicate cultures. Statistically significant differences with respect to untreated control (*p* < 0.05) are represented with one asterisk (*).

To determine the biocompatibility of the different fabricated nanoformulations, a toxicity study was performed using normal human cell lines with different degrees of cell proliferation, including fibroblast (MEF) and cells derived from breast (MCF10A), kidney (Hek 293T), and liver (HepG2). On the other hand, in order to assess the therapeutic potential of hybrid nanocarriers, a citotoxicy assay was carried out using the following cancer cell lines: A549 (lung cancer), MCF7 (breast cancer), MDA-MB-231 (triple negative breast cancer), and T84 (colon cancer). As a proof of concept, for these assays we selected PLGA@Ag_2_S_R1. As shown in Figure S13, for nanoparticle concentration lower than 50 μg/mL, a moderate toxic effect was appreciated only in the case of kidney cell line, while in the other cases, no cell toxicity was detected. Upon nanoparticle increase up to 100 μg/mL, a moderate cell toxicity was appreciated also for fibroblast and liver cell lines. These results indicate that PLGA@Ag_2_S_R1 nanoparticles show high biocompatibility, thus offering the possibility of biomedical applications. On the other hand, nanoparticles loaded with maslinic acid showed a good effectivity in all tested tumor cell lines, inducing a 50% proliferation inhibition within the 40–60 μg/mL dose range in all cases (Figure 7). 

In order to determine the colloidal stability of the nanosystems after the maslinic acid incorporation, we measured the Z-potential for PLGA@Ag_2_S_R1 and PLGA@Ag_2_S@maslinic in function of pH; these values are included in Figure S14. As it can be observed, in both cases, the isoelectric point values are lower than pH 3. These data demonstrate that the particle stability is not affected by the incorporation of the selected chemotherapeutic drug, this feature being very important for the colloidal stability of hybrid nanocarriers in the biological environment. 

### 3.6. In Vitro Drug Release Determination

**Figure 8:**
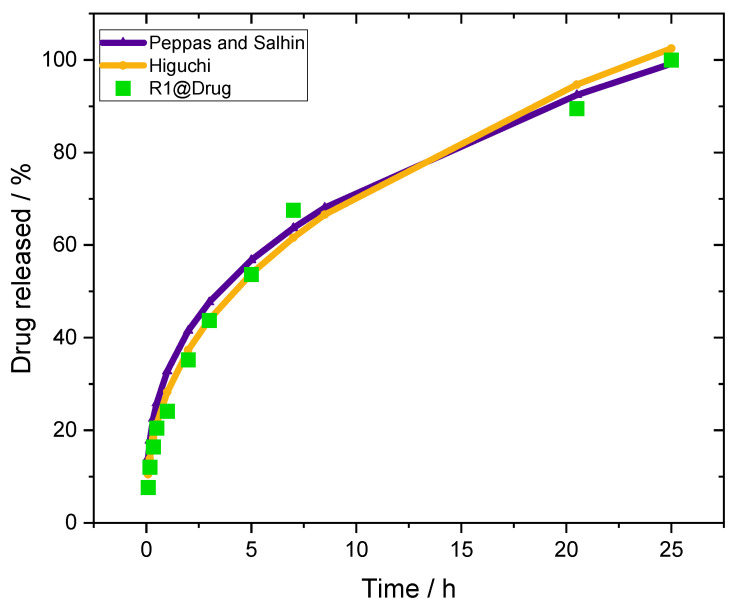
Kinetic release of the maslinic acid encapsulated into PLGA@Ag_2_S@SPION_R3 NPs (green dots). Higuchi model (orange line) and Peppas and Salhin model (blue line).

With the aim to generate new theragnostic devices to be applied in nanomedicine as drug delivery system [44,45,46], the hybrid PLGA@Ag_2_S@SPION_R2 NPs systems are here initially used as a nanocontainer for the encapsulation and release of bioactive molecules. As a chemotherapeutic drug, maslinic acid, a molecule of natural origin, extracted from the olive skin, with demonstrated cytotoxic activity, is here selected as an appropriate compound to test the encapsulation and release properties for PLGA@Ag_2_S@SPION_R2 NPs. For this aim, PLGA@Ag_2_S@SPION_R2 NPs were afforded by including maslinic acid (1% *w*/*w*) in the starting mixture previous to the electrospraying technique (see Supporting Information for details). As was detailed in Section 2.6, 3 mL of the PLGA@Ag_2_S@maslinic acid aqueous colloidal was centrifuged, extracted with chloroform, dried over MgSO_4_, and concentrated to dryness. HPLC analysis of the residue confirmed the absence of maslinic acid in the supernatant, and consequently, a trapping efficiency of maslinic acid into the PLGA@Ag_2_S@maslinic acid system of around 100%. The resulted in vitro release kinetic profile was fitted to a zero-order, first-order, Hixson–Crowell cube root model, Higuchi model (modified for diffusion swelled polymeric spherical systems), and Peppas and Salhin approximation model (for systems that present diffusion in a swelled polymeric matrix) [47,48,49], adding the relaxation effect of polymer chains during the diffusion process. As is shown in Figure 8, which represents the kinetic release of the maslinic acid encapsulated into PLGA@Ag_2_S@SPION_R3 nanosystem, the release profile pattern is characterized by an initial burst effect during the first 1–2 h. After this period, the sustained release was performed through diffusion and swelling processes. The Higuchi and the Peppas and Salhin models were the more accurate fit approximations according to the correlation coefficients. The *Higuchi* model describes a pure Ficknian diffusion-depending controlled release. It follows the equation with the best option in terms of correlation coefficients (R^2^), being 0.96 and 0.95 for Higuchi and Pepas and Shalhin models, respectively. The modified Higuchi model (blue line in Figure 8) presents an *n* coefficient with a value of 0.4, thus corresponding with a polymeric sphere swelled device, and a Higuchi release rate constant of 28.3, which represents a purely diffusion-controlled drug release. On the other hand, the Peppas and Salhin model (yellow line in Figure 8) presents two parts; the first added corresponds to the diffusion phenomenon (where K_1_ is the Ficknian diffusion constant), and the second added corresponds to the chain relaxation phenomenon (where K_2_ is the string relaxation constant). In this model, it is assumed that both phenomena are additive, however, the low value of K_2_ showed a poor contribution of the chain relaxation effect at 24 h of release. In Figure 8, Q_t_ is the quantity of the drug at a determined time, Q_∞_ is the quantity of the drug at t = infinite; K is the kinetic constant; T is time; and n and m are diffusion exponents. On the other hand, both models Higuchi and Peppas and Salhin fit perfectly with the experimental data, but also from a theoretical point of view. Both models share the idea that the release is controlled by diffusion, however, Peppas and Salhin introduce a new component based on chain relaxation-controlled release. In the end, the fact the chain relaxation factor is quantitatively smaller than the diffusion factor corroborates the Higuchi approximation of a purely diffusion-controlled drug release.

Higuchi model

$$Q_{t}=K_{H}\cdot t^{n}$$

*K_H_* = 28.3, *n* = 0.4, *R*^2^ = 0.98.

Peppas and Salhim model

$$\frac{Q_{t}}{Q^{\infty }}=K_{1}\cdot t^{m}+K_{2}\cdot t^{2m}$$

*K*_1_ = 32.7, *K*_2_ = 0.004, *m* = 0.34, *R*^2^ = 0.95.

## 4. Conclusions

In summary, due to the high interest in the fabrication of hybrid compounds for biomedical applications, here we obtain hybrid polymeric nanoparticles by the electrospraying technique for drug delivery applications. The hybrids systems were initially structured by PLGA as a biocompatible polymer in connection with PL Ag_2_S NPs. Three different synthetic routes were developed to generate hybrid PLGA@Ag_2_S NPs. Indeed, by using a coaxial injector during the electrospraying process, the Ag_2_S NPs were preferentially located within the PLGA matrix or in the periphery. The hybrid PLGA@Ag_2_S systems show PL emission intensities in the order of Ag_2_S@SH-PEG-OCH_3_ NPs when they are dispersed in a buffer medium. Importantly, PL emission and decay time results are in concordance with the quenching effect produced by water molecules to the PL emission of Ag_2_S NPs. By using the co-axial electrospraying methodology (route 4 and route 5), we were able to incorporate SPIONs into the hybrid colloidal system. Elemental mapping analysis demonstrated that the location of Ag_2_S NPs and SPIONs into the PLGA matrix can be controlled by means of the selected co-axial route. Magnetic characterization confirms that the presence of SPIONs inside the PLGA matrix provides the hybrid system with a superparamagnetic behavior, with a saturation magnetization value of 15 emu/g. Our methodology guarantees high sustainability because it generates stable polymeric nanoparticles in an easy and reproducible manner. We also incorporate maslinic acid into the hybrid system as a chemotherapeutic drug. Release investigations showed that the release profile fits with Higuchi and Pepas and Shalhin models. In vitro cytotoxicity analyses performed by using MCF10A, HEPG2, and HEK 293 cell lines showed good biocompatibility. In addition, high citotoxicity for some cancer cell lines (A549, MCF-7, MDA-MB-231 and T-84) was also demonstrated. Therefore, our methodology offers the possibility of being applied in the generation of a series of hybrid systems, which can be structured and coupled with other types of nanoparticles, or polymers, to be mainly used in nanomedicine. Specifically, PLGA@Ag_2_S@SPION NPs possess potential applications in magnetic hyperthermia and bioimaging combined with controlled drug release.

## Figures and Tables

**Table 1:** Composition in percentage and place of injection of the different routes.

	Denoted Name	Ag_2_S (%)	SPION (%)	Maslinic Acid (%)
Route 1	PLGA@Ag_2_S_R1	0.1	-	-
Route 1@Drug	PLGA@Ag_2_S@maslinic acid	0.1	-	0.3
Route 2	PLGA@Ag_2_S_R2	0.1 (in) *	-	-
Route 3	PLGA@Ag_2_S_R3	0.1 (ext) *	-	-
Route 4	PLGA@Ag_2_S@SPION_R4	0.1 (in) *	0.3 (ext) *	-
Route 5	PLGA@Ag_2_S@SPION_R5	0.1 (ext) *	0.3 (in) *	-

* in: internal injector. ext: external injector.

## Data Availability

The data presented in this study are available within the article and its Supplementary Materials.

## Supplementary Materials

The following supporting information can be downloaded at: https://www.mdpi.com/article/10.3390/pharmaceutics14010214/s1, Scheme S1: Schematic representation for the synthesis of PLGA@Ag_2_S_R2 NPs. Scheme S2: Schematic representation for the synthesis of PLGA@Ag_2_S_R3 NPs. Scheme S3: Schematic representation for the synthesis of PLGA@Ag_2_S@SPION_R5. Figure S1: Particle size distribution for (A) Ag_2_S@DDT and (B) Ag_2_S@SH-PEG-OCH3 QDs. Figure S2: Photoluminescence spectra of the Ag_2_S@DDT (black line) and Ag_2_S@SH-PEG-OCH3 (red line) NPs both in CHCl3 at a concentration of 1 mg/mL (exc. 800). Figure S3: Photograph of an aqueous colloidal dispersion for Ag_2_S@DDT (right) and Ag_2_S@SH-PEG-OCH3 (left) NPs both in water at a concentration of 1 mg/mL. Figure S4: Scanning electron microscopy (SEM) images of PLGA@Ag_2_S_R1 NPs. Figure S5: Energy-dispersive X-ray spectroscopy spectra of the included PLGA@Ag_2_S NPS showing the Ag and S regions. Figure S6: Fits for the exponential decay times for (A) PLGA@Ag_2_S_R1, (B) PLGA@Ag_2_S_R2, and (C) PLGA@Ag_2_S_R3 systems. Figure S7: Hydrodynamic diameters for PLGA@Ag_2_S_R1 hybrid systems obtained at different dispersion times: (A) 0 h (orange), 1 h (gray), 2 h (blue), (B) 5 h (purple), 8 h (green), 24 h (rose), and 48 h (blue). Figure S8: TEM images for hybrid PLGA@Ag_2_S_R2 particles performed at (A) 0 h, (B) 8 h, (C) 24 h, and (D) 48 h. Figure S9: Hydrodynamic diameters for PLGA@Ag_2_S_R2 hybrid systems obtained at different dispersion times: (A) 0 h (yellow), 1 h (orange) and 2 h (blue), and (B) 5 h (purple), 8 h (blue), 24 h (rose), and 48 h (green). Figure S10: TEM images for hybrid PLGA@Ag_2_S_R3 particles performed at (A) 0 h, (B) 8 h, (C) 24 h, and (D) 48 h. Figure S11: Magnetization as a function of the applied field at 5 and 300 K (filled and empty squares respectively) for SPION. Inset represents the low field region to evaluate the coercive fields at 5 and 300 K (straight and dashed lines respectively). Figure S12: Representative TEM images for hybrid PLGA@Ag_2_S@SPION_R4 particles: (A) freshly prepared and (B) after 6 weeks deposited on the TEM grid. PLGA@SPION@Ag_2_S_R5: (C) freshly prepared P and (D) after 6 weeks deposited on the TEM grid. Figure S13: Biocompatibility assay of cell lines treated with PLGA@Ag_2_S_R1. The cell lines were exposed to increasing concentrations of PLGA@Ag_2_S_R1 from 0.1 to 100 µg/mL for 72 h. Data represent the mean values ± SD of triplicate cultures. Statistically significant differences with respect to untreated control (p < 0.05) are represented with one asterisk (*). Figure S14: Z-Potential values in function of pH for (A) PLGA@Ag_2_S_R1 and (B) PLGA@Ag_2_S@maslinic systems.
