# Supplementary material for: Electrospraying as a Technique for the Controlled Synthesis of Biocompatible PLGA@Ag2S and PLGA@Ag2S@SPION Nanocarriers with Drug Release Capability
Source: Pharmaceutics. 2022 Jan 17;14(1):214. doi: 10.3390/pharmaceutics14010214 (PMC8781675; doi:10.3390/pharmaceutics14010214)
Supplement: Supplementary file 1 [file pharmaceutics-14-00214-s001.zip › pharmaceutics-1517329-supplementary.pdf]

pharmaceutics

Supplementary Materials: Electrospraying as technique for the controlled synthesis of biocompatible PLGA@Ag_2S and PLGA@Ag_2S@SPION nanocarriers with drug release capability

Alexis Alvear-Jiménez, Irene Zabala-Gutierrez, Yingli Shen, Gonzalo Villaverde, Laura Lozano Chamizo, Pablo Guardia, Miguel Tinoco, Beatriz Garcia-Pinel, José Prados, Consolación Melguizo, Manuel Ló-pez-Romero, Daniel Jaque, Marco Filice, Rafael Contreras-Cáceres

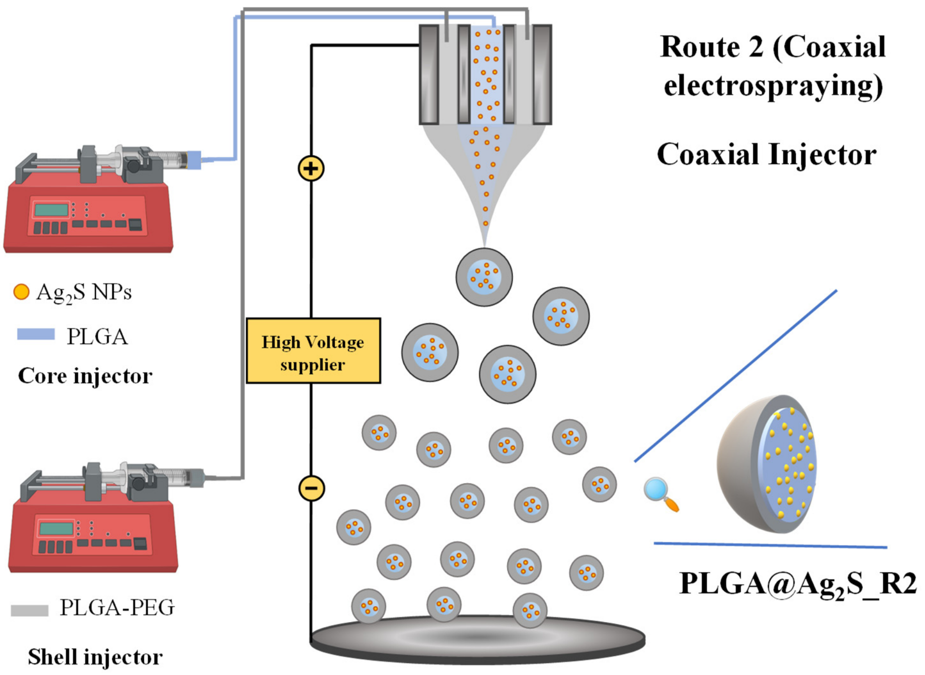

Scheme S1. Schematic representation for the synthesis of PLGA@Ag2S_R2 NPs

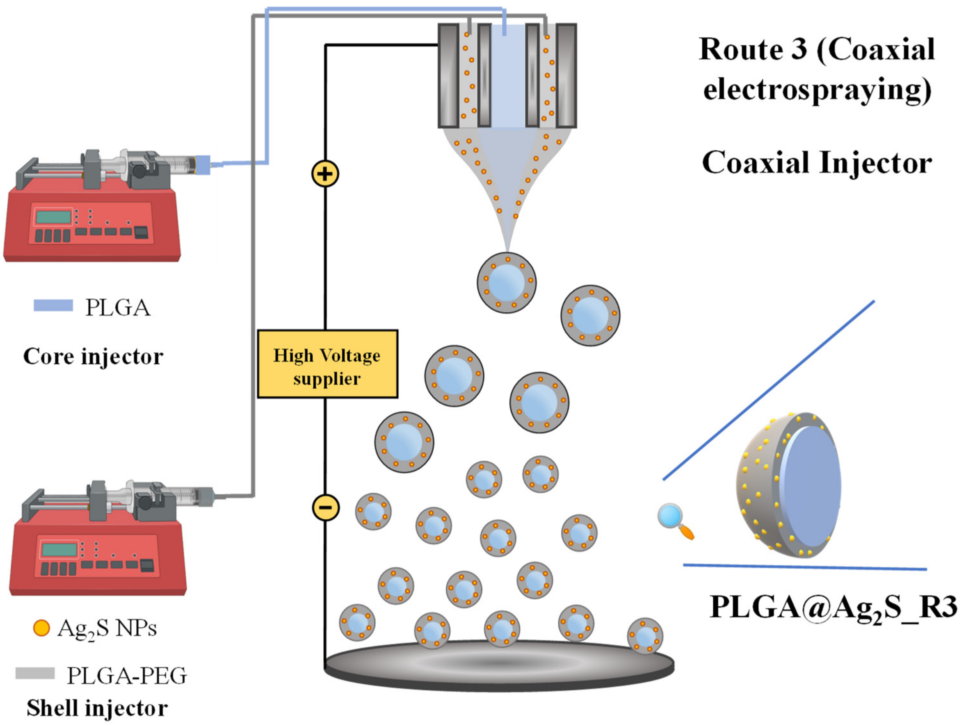

Scheme S2. Schematic representation for the synthesis of PLGA@Ag2S_R3 NPs.

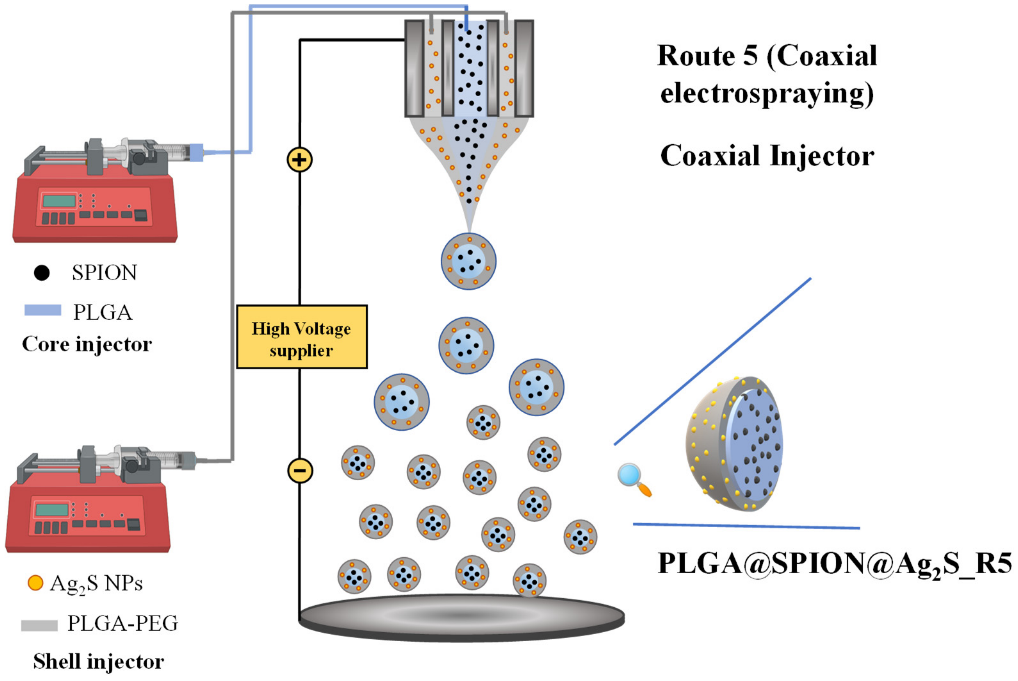

Scheme S3. Schematic representation for the synthesis of PLGA@Ag_2S@SPION_R5

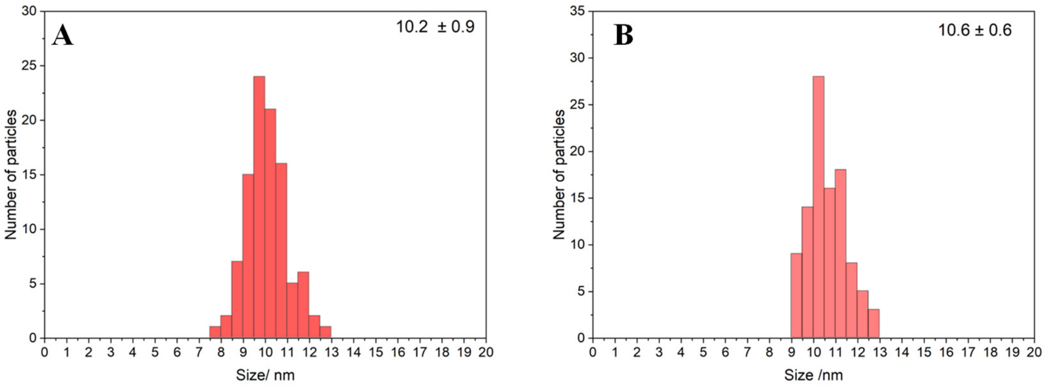

Figure S1. Particle size distribution for A) Ag_2S@DDT and B) Ag_2S@SH-PEG-OCH_3 QDs

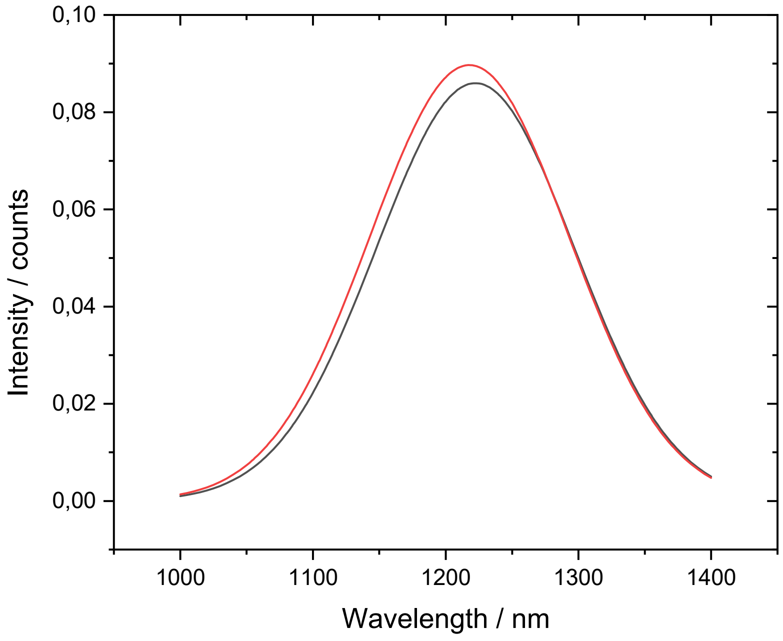

Figure S2. Photoluminescence spectra of the Ag_2S@DDT (black line) and Ag_2S@SH-PEG-OCH_3 (red line) NPs both in $CHCl_3$ at a concentration of 1 mg/mL (exc. 800).

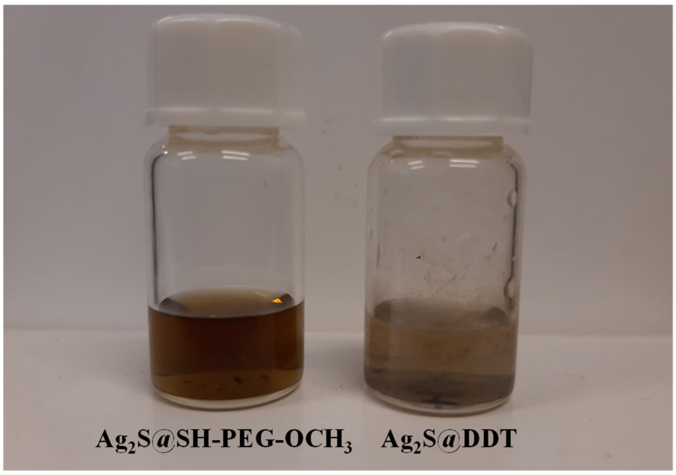

Figure S3. Photograph of a aqueous colloidal dispersion for Ag_2S@DDT (right) and Ag_2S@SH-PEG-OCH_3 (left) NPs both in water at a concentration of 1 mg/mL.

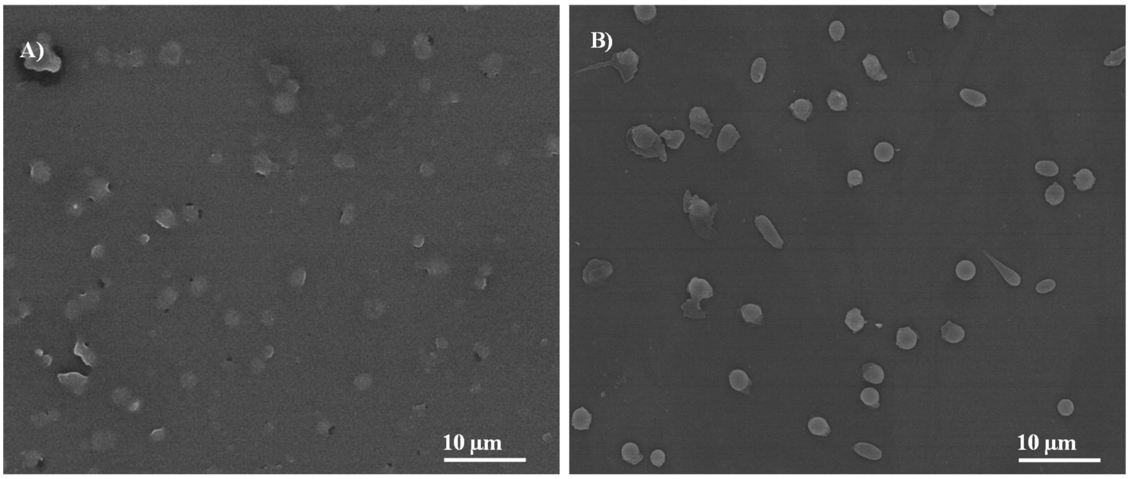

Figure S4. Scanning electron microscopy (SEM) images of PLGA@Ag_2S_R1 NPs

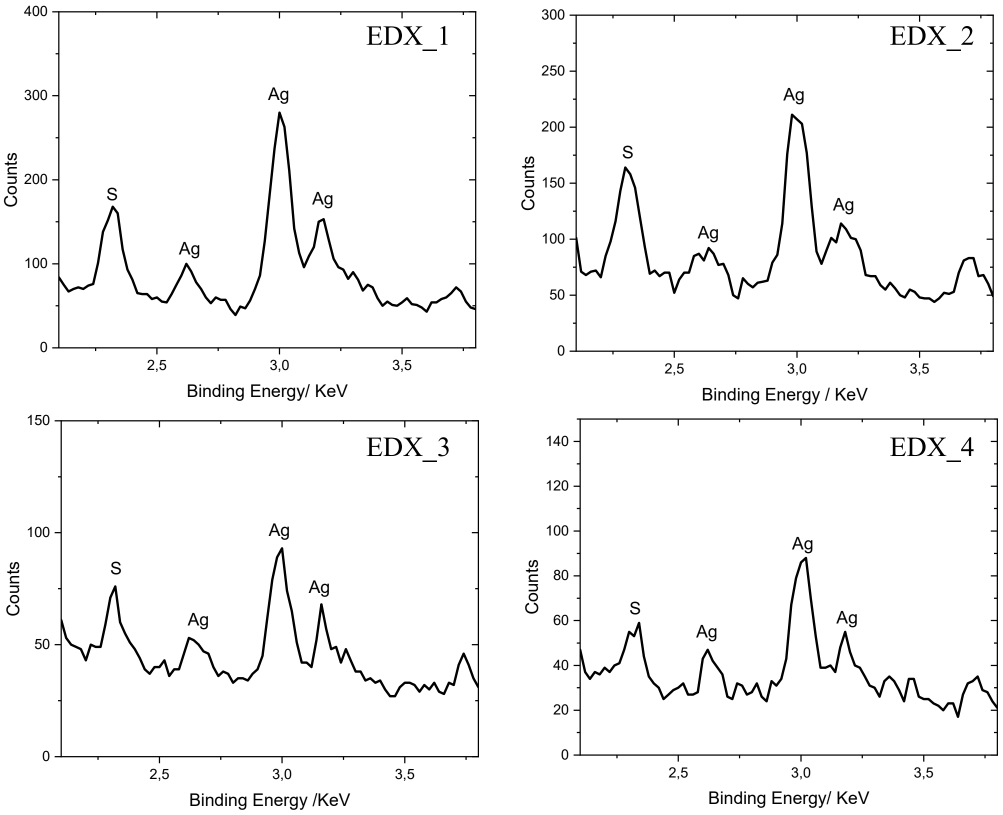

Figure S5. Energy-dispersive X-ray spectroscopy spectra of the included PLGA@Ag_2S NPS showing the Ag and S regions

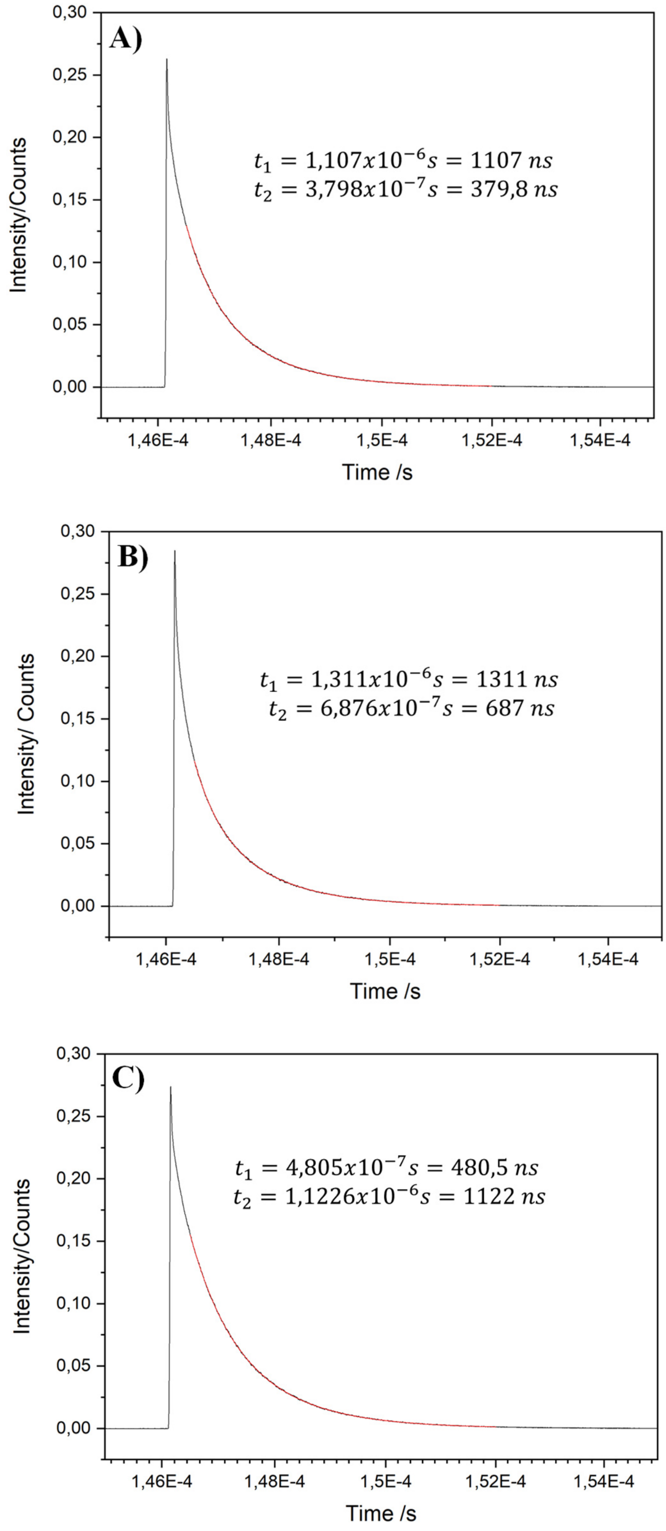

Figure S6. Fits for the exponential decay times for A) PLGA@Ag_2S_R1 B) PLGA@Ag_2S_R2 and C) PLGA@Ag_2S_R3 systems.

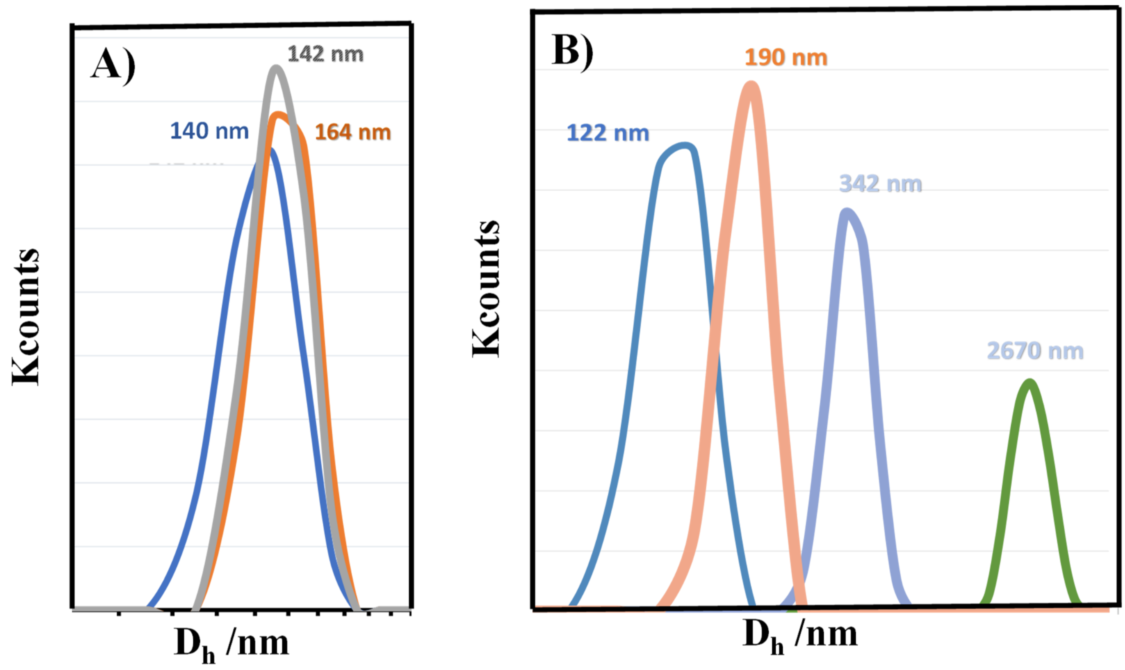

Figure S7. Hydrodynamic diameters for PLGA@Ag2S_R1 hybrid systems obtained at different dispersion times A) 0 h (orange), 1 h (gray) and 2 h (blue), and B) 5 h (purple), 8 h (green), 24 h (rose) and 48 h (blue).

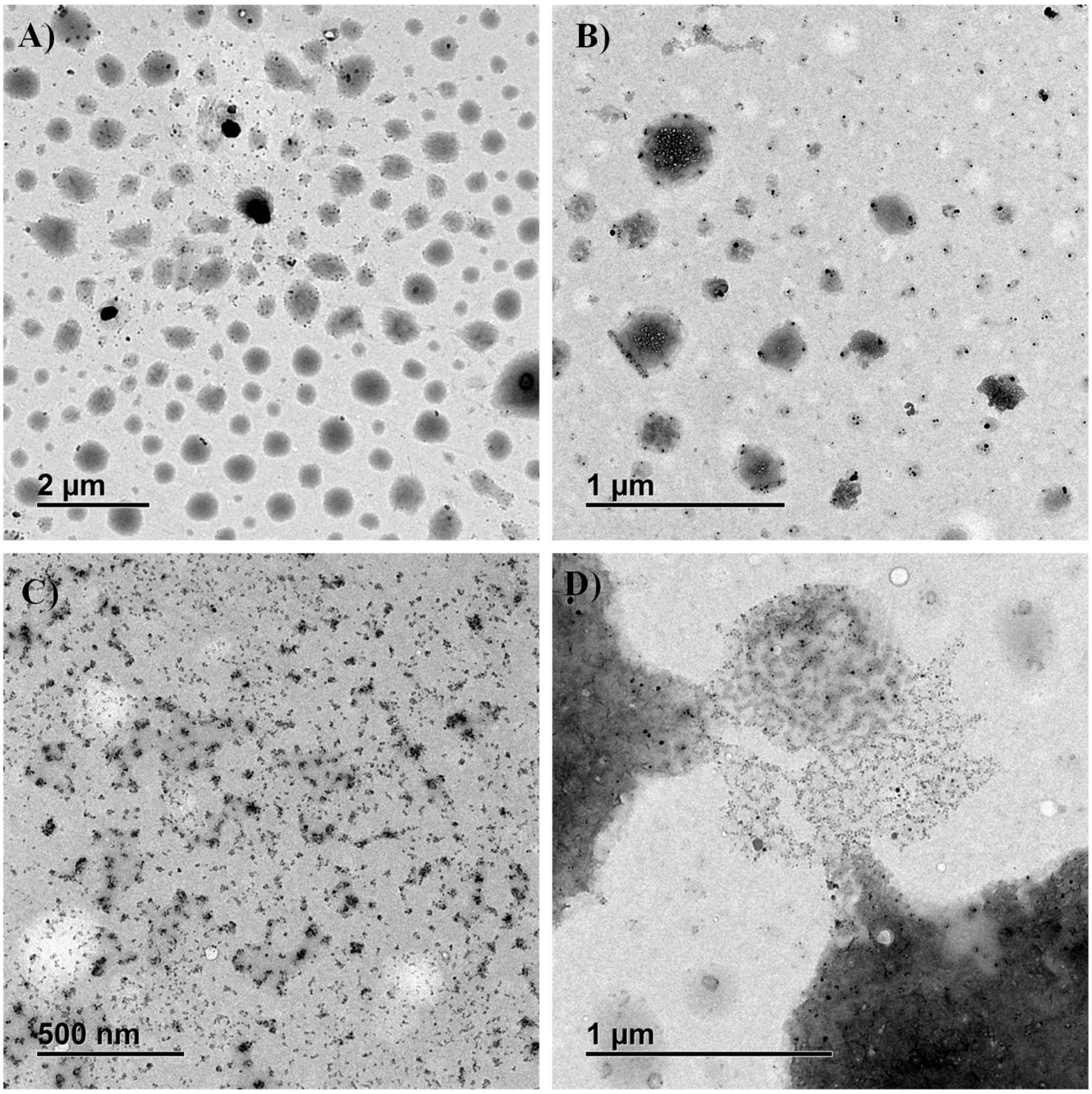

Figure S8. TEM images for hybrid PLGA@Ag2S_R2 particles performed at A) 0 h, B) 8 h, C) 24 h and D) 48 h

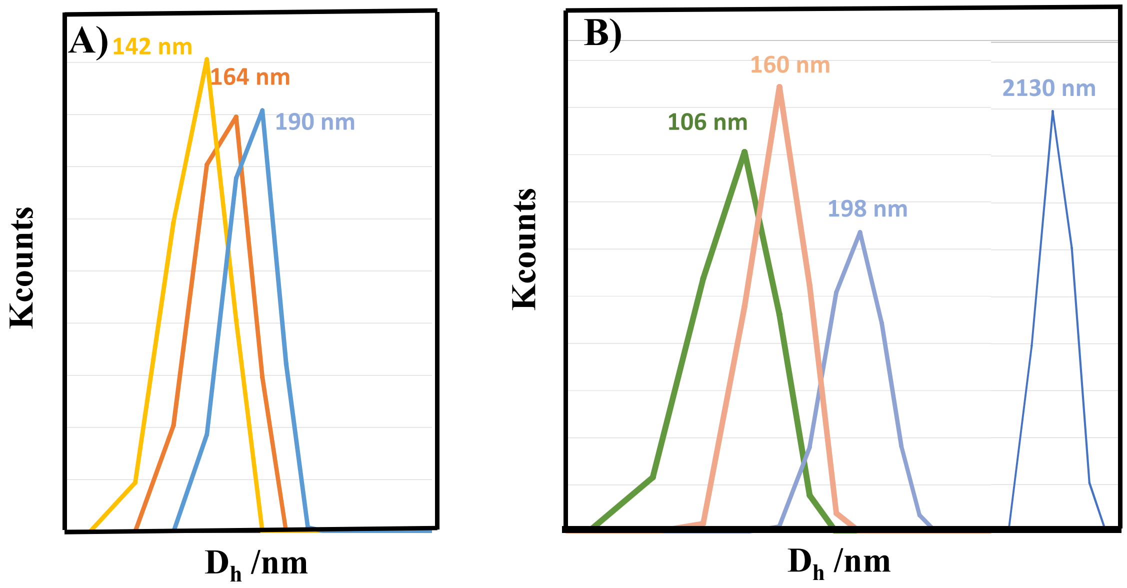

Figure S9. Hydrodynamic diameters for PLGA@Ag_2S_R2 hybrid systems obtained at different dispersion times A) 0 h (yellow), 1 h (orange) and 2 h (blue), and B) 5 h (purple), 8 h (blue), 24 h (rose) and 48 h (green).

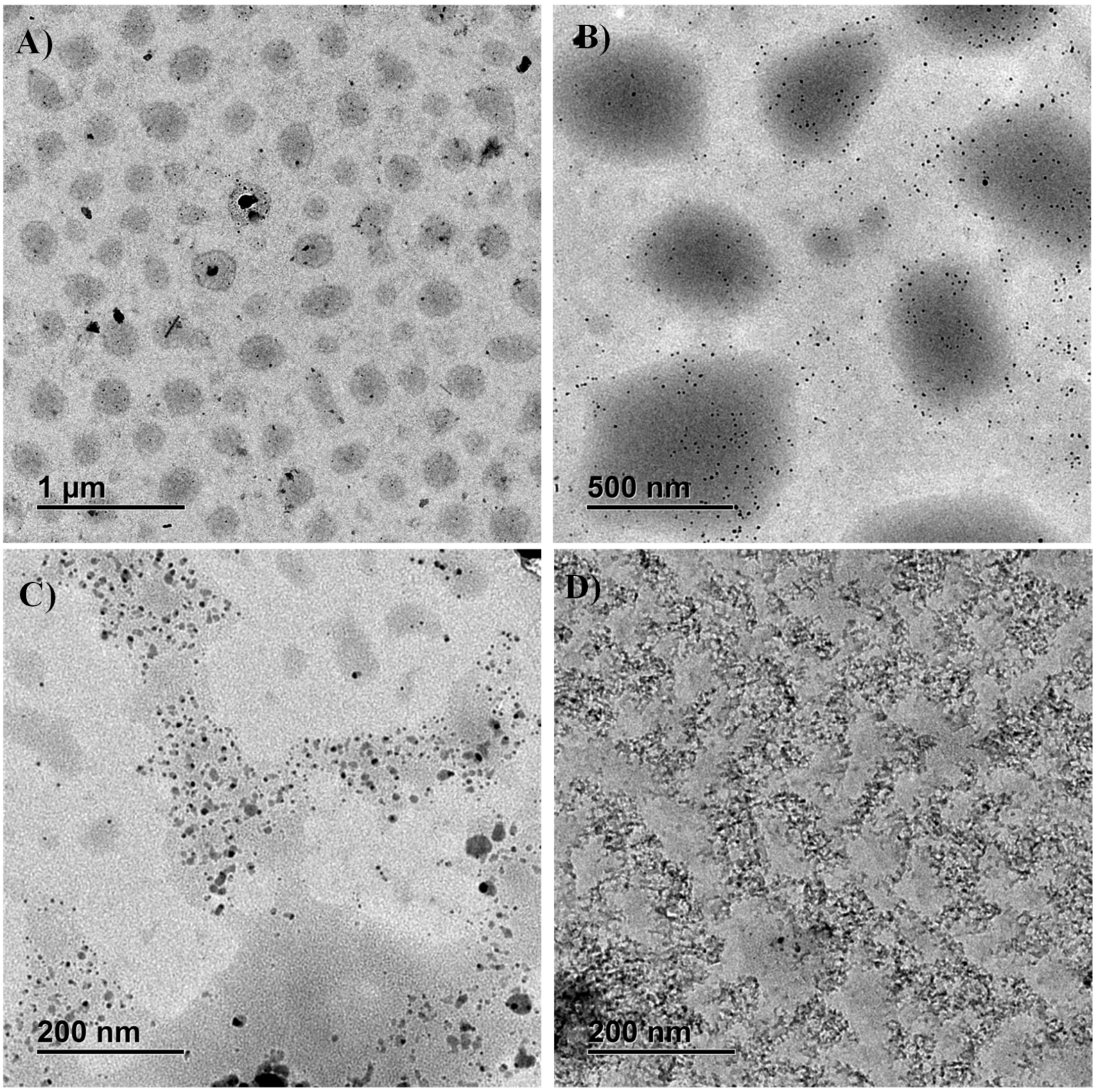

Figure S10. TEM images for hybrid PLGA@Ag2S_R3 particles performed at A) 0 h, B) 8 h, C) 24 h and D) 48 h

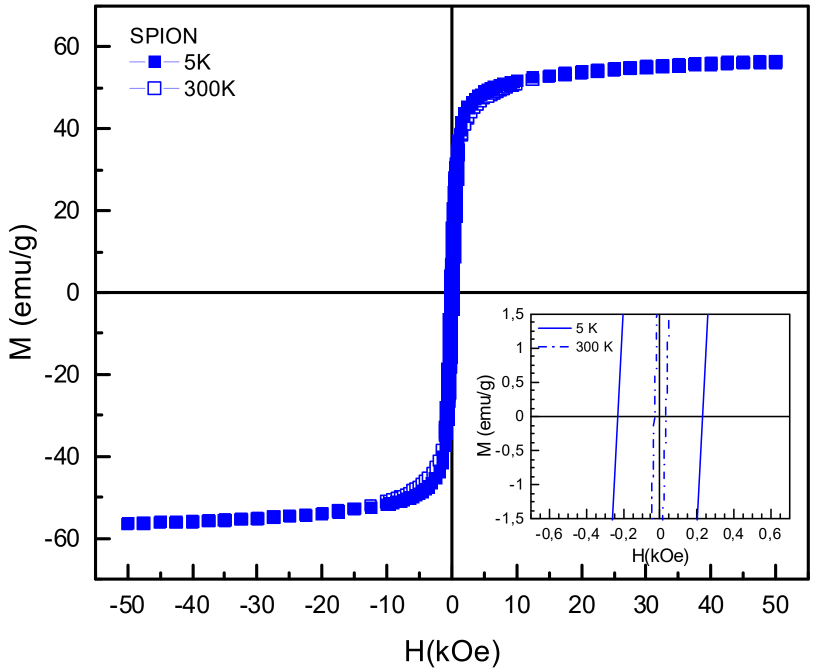

Figure S11: Magnetization as a function of the applied field at 5 and 300 K (filled and empty squares respectively) for SPION. Inset represents the low field region to evaluate the coercive fields at 5 and 300 K (straight and dashed lines respectively).

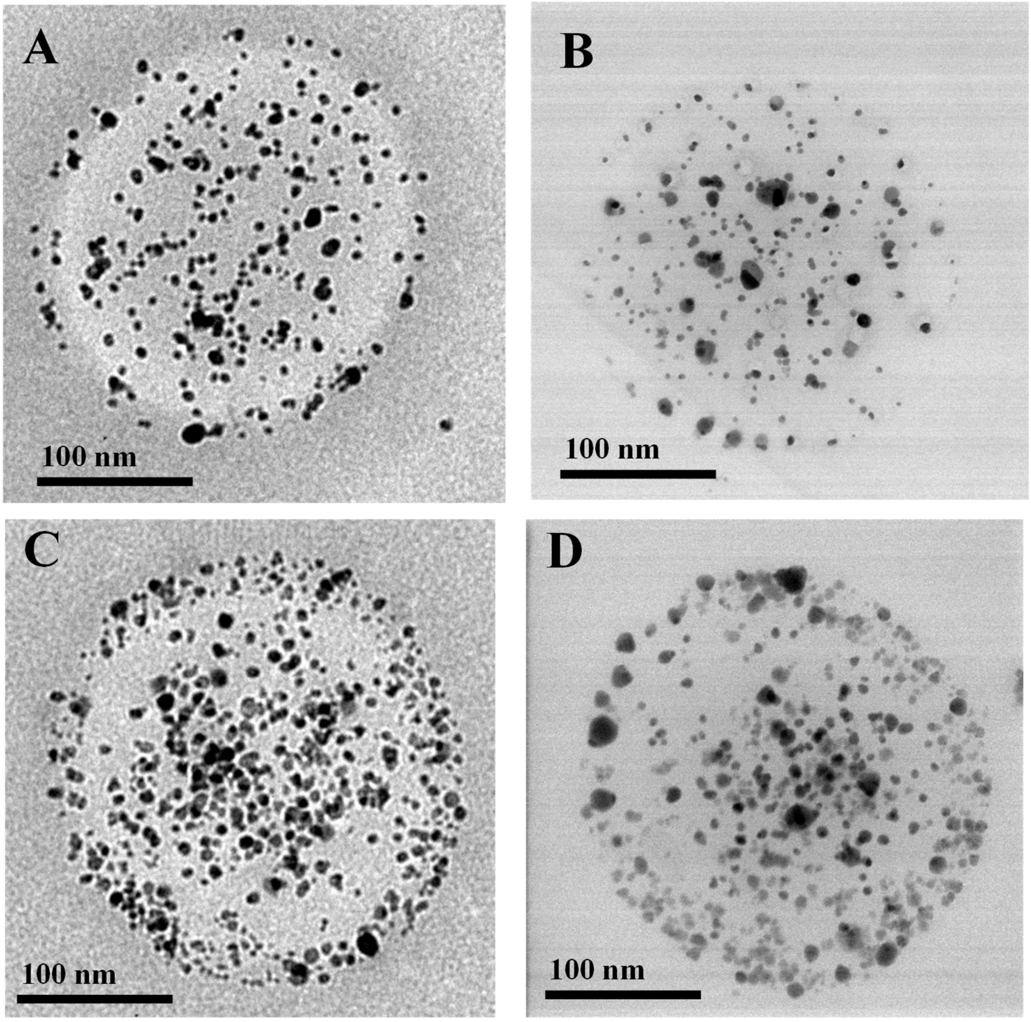

Figure S12. Representative TEM images for hybrid PLGA@Ag_2S@SPION_R4 particles A) freshly prepared and B) after 6 weeks deposited on the TEM grid. PLGA@SPION@Ag2S_R5 C) freshly prepared P and D) after 6 weeks deposited on the TEM grid.

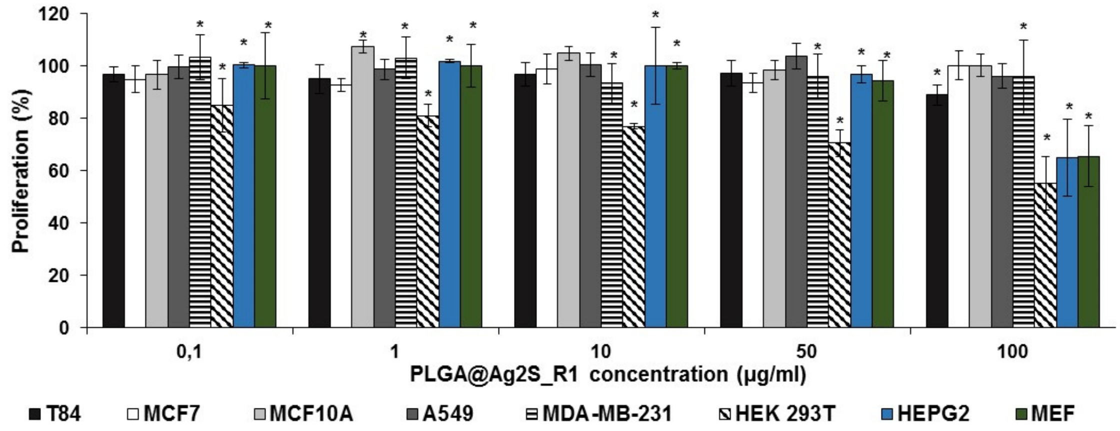

Figure S13. Biocompatibility assay of cell lines treated with PLGA@Ag2S_R1. The cell lines were exposed to increasing concentrations of PLGA@Ag_2S_R1 from 0.1 to 100 µg/mL for 72 h. Data represent the mean values ± SD of triplicate cultures. Statistically significant differences with respect to untreated control ($p < 0.05$) are represented with one asterisk (*).

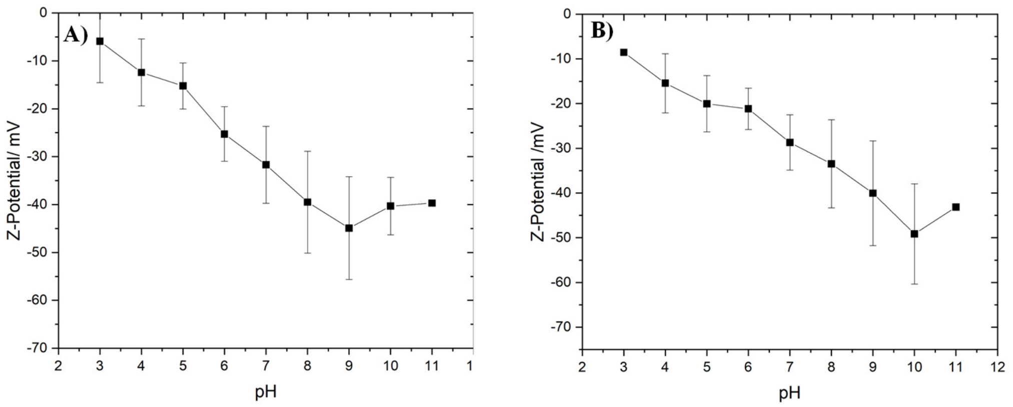

Figure S14. Z-Potential values in function of pH for A) PLGA@Ag_2S_R1 and B) PLGA@Ag_2S@maslinic systems.
